# [(CH_3_)Al(CH_2_)]_12_: Methylaluminomethylene (MAM‐12)

**DOI:** 10.1002/chem.202200823

**Published:** 2022-07-07

**Authors:** Georgios Spiridopoulos, Markus Kramer, Felix Kracht, Cäcilia Maichle‐Mössmer, Reiner Anwander

**Affiliations:** ^1^ Institut für Anorganische Chemie Eberhard Karls Universität Tübingen Auf der Morgenstelle 18 72076 Tübingen Germany; ^2^ Institut für Organische Chemie Universität Tübingen Auf der Morgenstelle 18 72076 Tübingen Germany

**Keywords:** aluminium, lutetium, methyl, methylene, titanium

## Abstract

The molecular structure of enigmatic “poly(aluminium‐methyl‐methylene)” (first reported in 1968) has been unraveled in a transmetalation reaction with gallium methylene [Ga_8_(CH_2_)_12_] and AlMe_3_. The existence of cage‐like methylaluminomethylene moieties was initially suggested by the reaction of rare‐earth‐metallocene complex [Cp*_2_Lu{(*μ*‐Me)_2_AlMe_2_}] with excess AlMe_3_ affording the deca‐aluminium cluster [Cp*_4_Lu_2_(*μ*_3_‐CH_2_)_12_Al_10_(CH_3_)_8_] in low yield (Cp*=C_5_Me_5_). Treatment of [Ga_8_(CH_2_)_12_] with excess AlMe_3_ reproducibly gave the crystalline dodeca‐aluminium complex [(CH_3_)_12_Al_12_(*μ*_3_‐CH_2_)_12_] (MAM‐12). Revisiting a previous approach to “poly(aluminium‐methyl‐methylene” by using a (C_5_H_5_)_2_TiCl_2_/AlMe_3_ (1 : 100) mixture led to amorphous solids displaying solubility behavior and spectroscopic features similar to those of crystalline MAM‐12. The gallium methylene‐derived MAM‐12 was used as an efficient methylene transfer reagent for ketones.

## Graphical abstract

**The next stage towards the cage**: [(CH_3_)Al(CH_2_)]_12_ (MAM‐12) is accessible in high yield through Ga/Al transmetalation with homoleptic gallium methylene [Ga_8_(CH_2_)_12_] and trimethylaluminium. MAM‐12 adopts a cage‐like structure, similar to the structural motifs proposed for the elusive methylaluminoxane [(CH_3_)Al(O)]_*n*_ (MAO), featuring three distinct aluminium coordinations. MAM‐12 is insoluble in aliphatic/aromatic solvents and disaggregates to MAM‐3 and [Me_2_Al‐CH_2_‐AlMe_2_] in a coordinating solvent like THF as suggested by a DOSY NMR experiments.

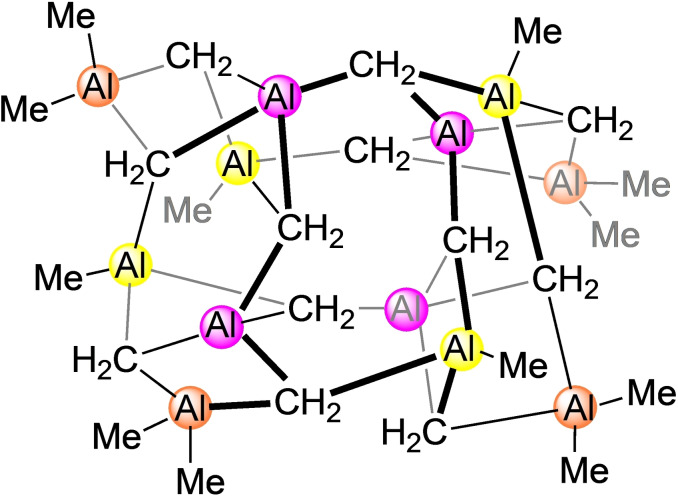

## Introduction

**Figure 1:**
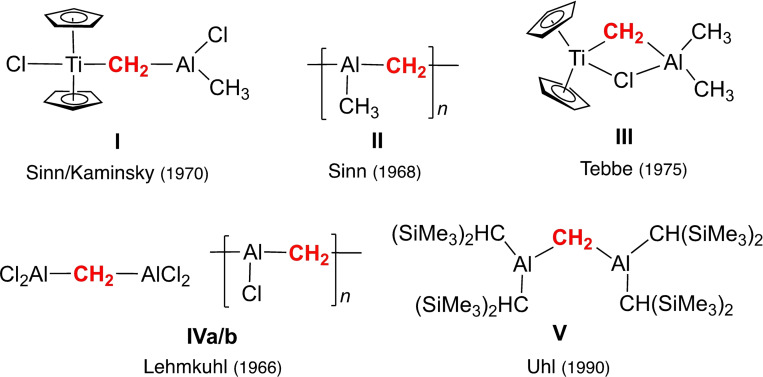
Milestones in aluminium methylene chemistry.

The interplay of early d‐transition organometallics and organoaluminium compounds has branched out into two major fields of organometallic research, Ziegler–Natta polymerization catalysis^[1]^ and, subsequently, metal alkylidene chemistry.^[2]^ In particular, the binary system (C_5_H_5_)_2_TiCl_2_/AlMe_3_, initially probed as a model to elucidate reaction pathways and active species in Ziegler's Mischkatalysatoren,^[3]^ strongly influenced the development of discrete metal alkylidene derivatives[^2^, ^4^, ^5^] and their use in olefination reactions^[6]^ and catalytic olefin metathesis.^[7]^ Initial investigations of the (C_5_H_5_)_2_TiCl_2_ reaction by Sinn and Kaminsky (1970) proved the formation of [Ti‐CH_2_‐Al] moieties (proposed structure **I**, Figure 1) and concomitant methane by α‐H abstraction.^[8]^ Solvent‐free mixtures of (C_5_H_5_)_2_TiCl_2_/AlMe_3_ (1 : 100) slowly (100–350 h) afforded a grayish solid analyzed as “poly(aluminium‐methyl‐methylene)” (**II**, Figure 1), which was soluble in THF.^[9]^ Apparently, the synthesis of **II** features the Tebbe reagent [(C_5_H_5_)_2_Ti(*μ*‐Cl)(μ‐CH_2_)AlMe_2_] (**III**, Figure 1) as an intermediate species. Tebbe could selectively synthesize his compound in 1974 using (C_5_H_5_)_2_TiCl_2_/AlMe_3_ in a 1 : 2 ratio (toluene, RT, 60 h).[^2f^, ^5^] More recently, the solid‐state structure of the Tebbe reagent could be elucidated by X‐ray diffraction (XRD) analyses.[^10^, ^11^] Interestingly, a more detailed mechanistic investigation of the system (C_5_H_5_)_2_TiCl_2_/AlMe_3_ by Grubbs and co‐workers from 1984 also pointed to the formation of red toluene‐insoluble species (“poly‐TiCH_2_Al”) when **III** was allowed to stand with excess AlMe_3_ or in neat AlMe_3_ for long reaction times.^[12]^ Discrete [Cl_2_Al‐CH_2_‐AlCl_2_] (**IVa**) and polymeric chloridoaluminomethylene species **IVb** (Figure 1), reminiscent of polymeric **II** were obtained in 1966 by Lehmkuhl and Schäfer from Al/CH_2_Cl_2_ mixtures.[^13^, ^14^] Years later in 1990, Layl and Uhl converted [Cl_2_Al‐CH_2_‐AlCl_2_] into the first alkyl aluminomethylene [R_2_Al‐CH_2_‐AlR_2_] (**V**, R=CH(SiMe_3_)_2_; Figure 1) by salt metathesis with lithium bis(trimethylsilyl)methyl.^[15]^ It is also noteworthy that partial pyrolysis of a concentrated solution of 14.8 g AlMe_3_ in hexane at 175–180 °C in autoclaves produced 2.5 g of a white solid which was analyzed through reaction with heavy water as mixed aluminium [methyl‐methylene‐methine‐carbide].^[16]^

Our research in this field was triggered by the “Lanthanide Model in Ziegler–Natta Polymerization” proposed by P. Watson in 1982^[17]^ and the topic “Rare‐Earth Metals and Aluminum Getting Close in Ziegler‐type Organometallics” is the strategy we have embarked on during the past 25 years.^[18]^ Accordingly, the interplay of rare‐earth metals and group 13 compounds not only emerged in thermodynamically very stable hetero‐bimetallics like homoleptic [Ln(AlMe_4_)_3_]^[19]^ but also in the targeted formation of isolable [Ln‐CH_2_‐Al]^[20]^ and [Ln‐CH−Al] moieties.^[21]^ More recently, the Ln/group 13 approach paved the way to unprecedented group 13 organometallics: the pseudo‐catalytic reaction of [Cp*_2_Ln{(*μ*‐Me)_2_GaMe_2_}] (Ln=Y, Lu; Cp*=C_5_Me_5_) with excess GaMe_3_ at elevated temperatures afforded homoleptic gallium methylene [Ga_8_(CH_2_)_12_].^[22]^ Noteworthy, the isolation of dodecametallic intermediate [(Cp*_2_Lu)_3_(*μ*_3_‐CH_2_)_6_Ga_9_(*μ*‐CH_2_)_9_] gave insight into the mechanism of such methylidene formation. Aiming at a wider applicability of this methyl degradation (α‐H abstraction) approach, the present study targets the activation of trimethylaluminium.

## Results and Discussion

**Scheme 1:**
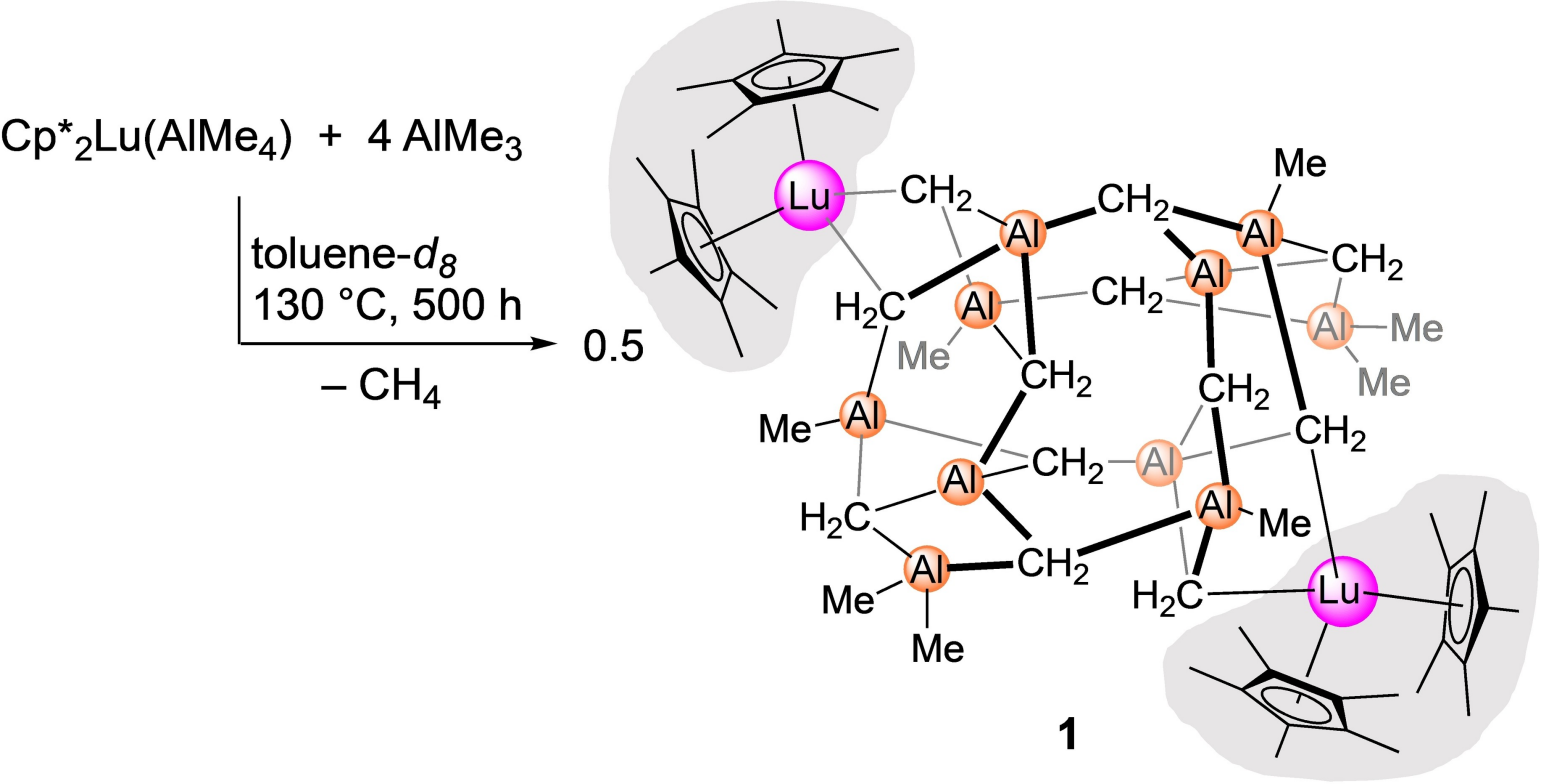
Synthesis of complex [Cp*_4_Lu_2_(μ_3_‐CH_2_)_12_Al_10_(CH_3_)_8_] (**1**), which was obtained in very low crystalline yield; a larger quantity of single‐crystalline **1** could not be obtained, thus impeding a more comprehensive characterization.

**Figure 2:**
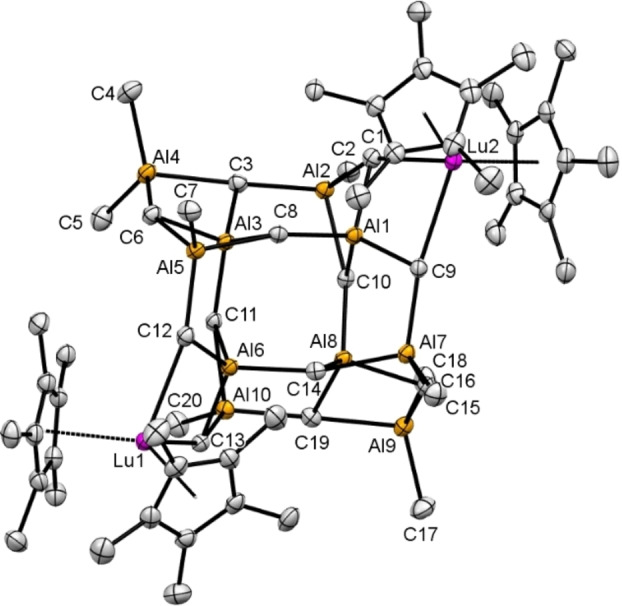
Crystal structure of coμplex **1**. Atomic displacement parameters are set at 50 % probability, and hydrogen atoms are omitted for clarity. For selected interatomic distances and angles, see the Supporting Information.

Treatment of [Cp*_2_Lu{(*μ*‐Me)_2_AlMe_2_}]^[23]^ with 4 equiv. AlMe_3_ at 130 °C in [D_8_]toluene generated methane (Figure S1 in the Supporting Information) and produced a few colorless crystals of [Cp*_4_Lu_2_(*μ*_3_‐CH_2_)_12_Al_10_(CH_3_)_8_] (**1**, Scheme 1). The XRD analysis of compound **1** revealed an asymmetric cage‐like structural motif (Figure 2). Two opposite corners of the molecule are occupied by [Cp*_2_Lu] metallocene units which are bridged by two *μ*_3_‐methylidene groups to the {Al_10_} entity. The Lu−C_methylene_ distances range from 2.499(4) to 2.630(4) Å (avg. 2.568 Å), being significantly shorter than those in the gallium congener [Cp*_6_Lu_3_(*μ*_3_‐CH_2_)_6_Ga_9_(CH_2_)_9_] (avg. 2.614 Å).^[22]^

The distinct coordination environments of the aluminium atoms featuring two Al(*μ*_3_‐CH_2_)_2_(CH_3_)_2_, four Al(*μ*_3_‐CH_2_)_3_(CH_3_), and four Al(*μ*_3_‐CH_2_)_4_ tetrahedra are striking. The Al−C(Me) distances average 1.958 Å, similar to the terminal aluminium methyls in Al_2_Me_6_ (avg. 1.9556 Å).^[24]^ As expected, the Al−C(*μ*_3_‐CH_2_) distances are longer for the Al(*μ*_3_‐CH_2_)_4_ moieties (avg. 2.011 Å) compared to the Al atoms that carry two and three methylene groups (avg 1.952 Å). For comparison, the Al−C(*μ*_2_‐CH_2_) distances in Uhl's trigonal planar [(AlR_2_)_2_(*μ*‐CH_2_)] (R=CH(SiMe_3_)_2_,^[14]^ and tetranuclear heteroadamantane [Al_4_(*μ*‐CH_2_)_2_Cl_4_R_4_]^[25]^ amount to 1.938(1) and 1.959(2) Å, respectively. The ^1^H NMR spectrum of **1** in [D_8_]THF shows a signal at 1.87 ppm for the C_5_ *Me*_5_ ancillary ligands. Separate resonances at −0.96, −0.99, −1.16, and −1.81 ppm are assigned to [AlC*H*_3_], [LuC*H*_2_Al], and [AlC*H*_2_Al] moieties (Figure S2), respectively, thus suggesting the absence of CH_2_/CH_3_ exchange processes. Such a rigid arrangement is in agreement with the observations made for [Tp^*t*Bu,Me^La(*μ*_3_‐CH_2_){(*μ*_2_‐Me)AlMe_2_}_2_]^[20a]^ and [(PNP)Sc(*μ*_3_‐CH_2_){(*μ*_2_‐Me)AlMe_2_}_2_] having revealed separate signals for C*H*_2_/C*H*_3_ at ambient temperature.^[20b]^ Unfortunately, a scale‐up reaction using [Cp*_2_Lu(AlMe_4_)] and excess trimethylaluminium did not lead to the extrusion of a methylaluminomethylene (MAM) species [(CH_3_)_x_Al_y_(*μ*_3_‐CH_2_)_z_] or homoleptic aluminium methylene, as observed in the gallium methylene study.^[22]^

**Scheme 2:**
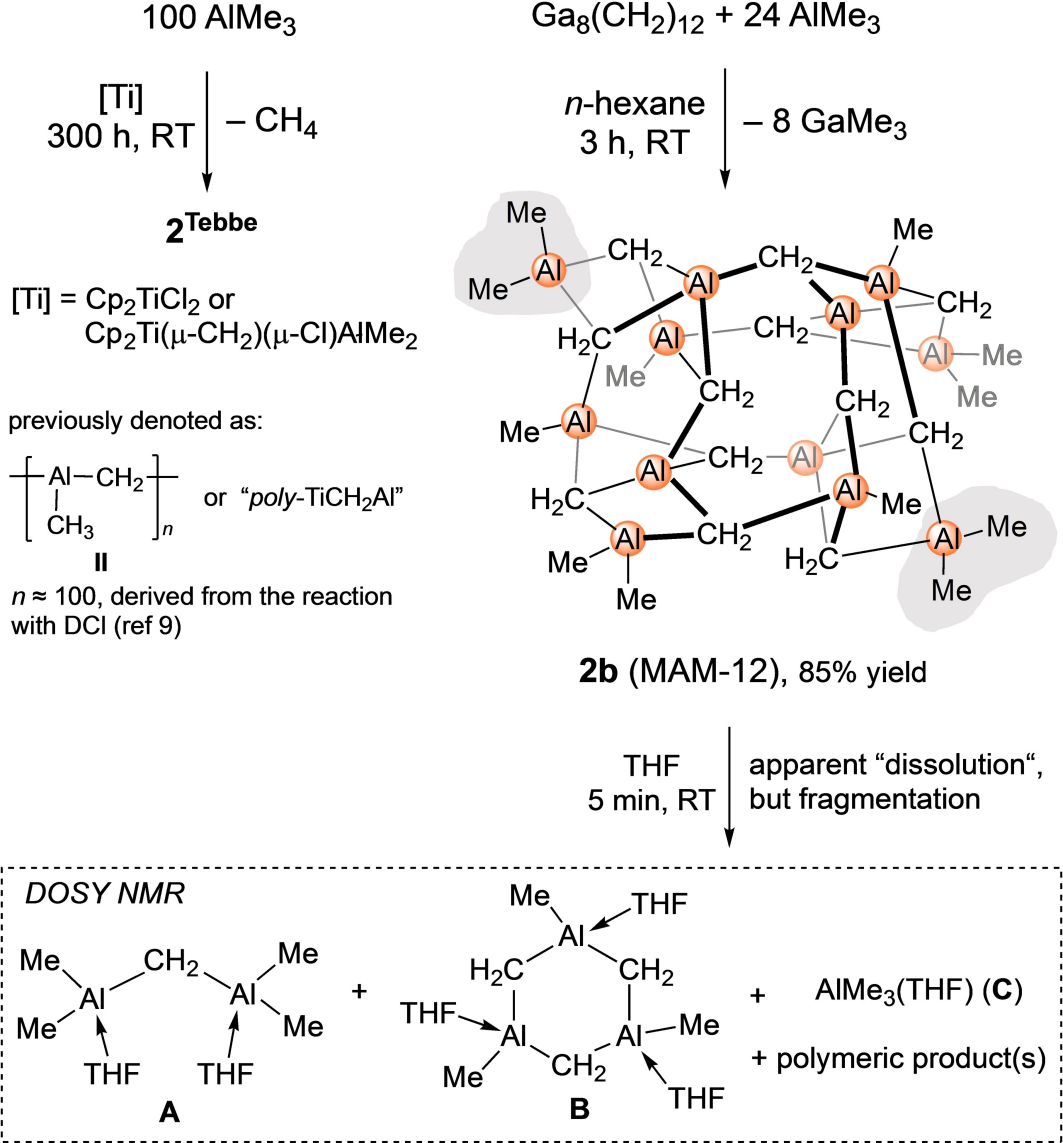
Synthesis of **2** ^Tebbe^ and dodecametallic [(CH_3_)_12_Al_12_(μ_3_‐CH_2_)_12_] (**2 b**), along with fragmentation of **2 b** in THF.

In the quest for alternative approaches to putative MAM species, we sought to reinvestigate the synthesis reported by Sinn et al.^[8]^ As originally described, the reaction of dichlorido titanocene (C_5_H_5_)_2_TiCl_2_ or Tebbe's [(C_5_H_5_)_2_Ti(*μ*‐CH_2_)(*μ*‐Cl)Al(CH_3_)_2_] and neat AlMe_3_ (100 equiv.) afforded aluminium‐methyl‐methylene (**2** ^Tebbe^; Scheme 2, left). Purification of **2** ^Tebbe^ by the Soxhlet method (benzene, 3 d) left a reddish powder (not gray as described by Sinn for **II**,^[9]^ but red as mentioned by Grubbs for “poly‐TiCH_2_Al”).^[12]^ The color indicated minor contamination with a titanium(III) species which was confirmed by EPR and ICP‐OES analysis (Ti 0.33 %; see the Supporting Information Figure S5). Compound **2** ^Tebbe^ is insoluble in aliphatic and aromatic solvents but dissolves slightly in THF. The ^1^H NMR spectrum of **2** ^Tebbe^ in [D_8_]THF shows a signal pattern similar to compound **1** with resonances of the Al‐C*H*_2_/C*H*_3_ moieties detectable at −0.96, −0.99, −1.03 and −1.81 ppm (Figure S3).

**Figure 3:**
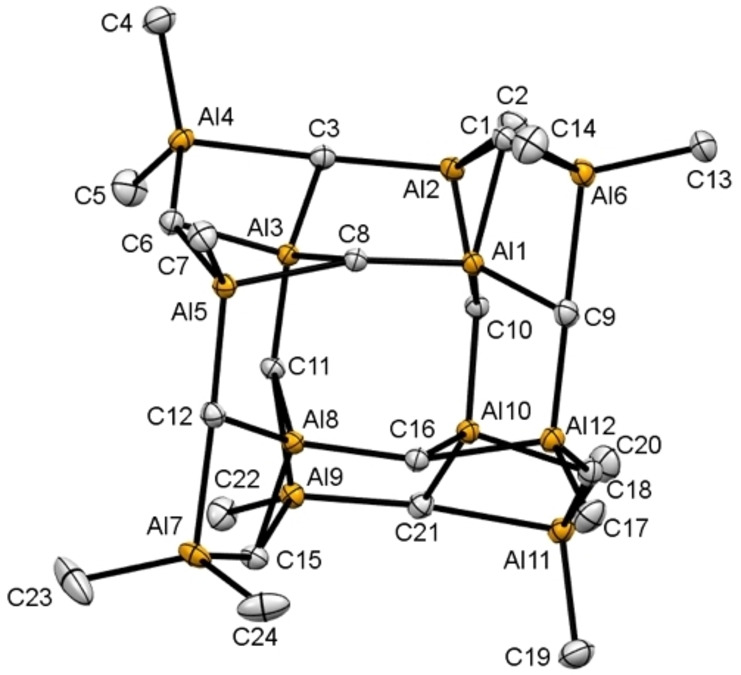
Crystal structure of [(CH_3_)_12_Al_12_(μ_3_‐CH_2_)_12_] (**2 b**, MAM‐12). The atomic displacement parameters are set at 50 % probability. All hydrogen atoms except those for C19 were located in the difference Fourier maps, but are omitted for clarity. For a representation of all atoms, selected interatomic distances and angles, see the Supporting Information.

With the homoleptic gallium methylene [Ga_8_(CH_2_)_12_] in hands, we next examined the feasibility of a Ga/Al transmetalation.^[26]^ Treatment of pale yellow suspensions of [Ga_8_(CH_2_)_12_] in non‐coordinating solvents (benzene or *n*‐hexane) with excess AlMe_3_ (12 or 24 equiv.) led to a steady decolorization of the mixture and the temporary generation of a clear solution. After stirring the mixtures for 2–3 h at ambient temperature colorless precipitates had formed (Scheme 2, right). The obtained powders **2 a** (12 equiv. AlMe_3_) and **2 b** (24 equiv. AlMe_3_) feature limited solubility and the ^1^H NMR spectra in [D_8_]THF revealed signal patterns similar to treddish **2** ^Tebbe^ and compound **1**. Consequently, this encourages the assumption that the two protocols depicted in Scheme 2 generated similar compounds/materials. Fortunately, single crystals of **2 a**/**2 b** were obtained from microscale reactions and crystallization at 70 °C. The XRD analysis of [(CH_3_)_12_Al_12_(*μ*_3_‐CH_2_)] (**2 b**, MAM‐12) revealed again a cage‐like structural motif similar to compound **1** (Figure 2). In **2 b** the two peripheral [Cp*_2_Lu]^+^ units in **1** are displaced by [AlMe_2_]^+^ moieties (see the gray areas depicted in Figures 2 and 3).

The aluminium coordination environments in fully Ga/Al‐exchanged compound **2 b** now each comprise four Al(*μ*_3_‐CH_2_)_2_(CH_3_)_2_, Al(*μ*_3_‐CH_2_)_2_(CH_3_)_2_, and Al(*μ*_3_‐CH_2_)_4_ tetrahedra. Incompletely Ga/Al‐exchanged compound **2 a** displays a partial occupancy for the four M(CH_3_)_2_ positions as a special case of substitutional disorder. Accordingly, the group 13 metal M was refined on the same position in a ratio Al/Ga=0.66:0.34 (Figure S27). The Al−C(Me) distances in **2 b** average 1.952 Å, and are thus similar to those in compound **1**. The average Al−C(μ‐methylene) distances of 2.011 Å also match those in compound **1** and other clusters containing Al−CH_2_ moieties (e. g., [La_4_Al_8_(C)(CH)_2_(CH_2_)_2_(CH_3_)_22_(toluene)].^[27]^ Other cage‐like organo‐{Al_12_} clusters include low‐valent icosahedral K_2_[Al_12_ *i*Bu_12_]^[28]^ and ellipsoidal Li[Al_12_{N(SiMe_3_)_2_}_8_].^[29]^ It is also interesting to note that (CH_3_)_18_Al_12_O_9_ cage clusters have been suggested (from experimental studies and DFT calculations) as a model for methylaluminoxane (MAO) cocatalyst solutions, employed in olefin polymerization.^[30]^

Compound **2 b** decomposes at 380 °C, is insoluble in aliphatic and aromatic solvents but apparently “dissolves” in THF, in accordance with the solution behavior of **II**.^[8]^ The ^1^H NMR spectrum of **2 b** in [D_8_]THF shows four resonances at −0.97, −1.00, −1.04 and −1.82 ppm. The slightly broadened high‐field signal at −1.82 ppm can be assigned to Al−C*H*_2_ moieties and the remaining signals represent terminal aluminium methyl groups. The proton signal of trimethylaluminium in [D_8_]THF is detected at −1.02 ppm. For further comparison, the methylene signal of 3‐coordinate [{(Me_3_Si)_2_HC}_2_Al‐C*H*_2_‐Al{CH(SiMe_3_)_2_}_2_] (**V**) was reported as −0.50 ppm (in C_6_D_6_) while the C*H*_2_ signals of alkynyl ate complexes of **V** involving 4‐coordinate aluminium centers were found significantly shifted upfield in the range −1.03 to −1.76 ppm ([D_8_]THF).^[31]^ The ^13^C NMR spectrum of **2 b** in [D_8_]THF shows also four signals for aluminium‐bonded carbon atoms at −4.79, −6.58, −7.63, and −9.20 ppm, while the ^27^Al NMR spectrum in [D_8_]THF gave three signals in the range 160–127 ppm. ^13^C‐DEPT135, ^1^H,^13^C HSQC, and ^1^H,^13^C HMBC NMR spectra were recorded as well as a VT ^1^H NMR study in the range −80 °C to +80 °C (ruling out any dynamic behavior or exchange processes) carried out to further elucidate the behavior of **2 b** in solution. Particularly enlightening proved the ^1^H,^13^C HMBC NMR spectrum (Figure S15) combined with a ^1^H DOSY NMR experiment (Figures S20–S22). Clearly, the experimental data obtained suggest fragmentation of the dodecametallic cluster **2 b** into at least three species with molecular masses matching those of putative {[(THF)Me_2_Al‐CH_2_‐AlMe_2_(THF)]} (**A**), {[MeAl(CH_2_)(THF)]_3_} (**B**), and {AlMe_3_(THF)} (**C**) (Scheme 2, Figure S20). Species **A** is reminiscent of [{(Me_3_Si)_2_HC}_2_Al‐C*H*_2_‐Al{CH(SiMe_3_)_2_}_2_] (**V**). **V**‐type complexes have been previously also reported for the smaller terminal alkyl ligands methyl and ethyl, but have remained elusive. Compounds [Me_2_Al‐CH_2_‐AlMe_2_] and [Et_2_Al‐CH_2_‐AlEt_2_] were described as thermally labile and unstable in hydrocarbons decomposing to AlR_3_ and (polymeric) methylene‐bridged aluminium species (*δ*CH_2_: −1.88 to −2.11 ppm), but could be stabilized in the presence of diethyl ether.[^15^, ^32^] Desolvated trimetallic species **B** ({[MeAl(CH_2_)]_3_}) and decomposition products thereof could be detected by EI mass spectrometry. Monometallic AlMe_3_(THF) (**C**) could originate from the excessive AlMe_3_ used for the synthesis of **2 b** (Figures S7–S9) or dismutation of **2 b** when fragmentation occurred in THF. The latter dismutation reaction would also involve the formation of a methyl‐depleted (methylene‐rich) organoaluminium polymer which could not be identified. The likely occurrence of a respective dismutation reaction seems supported by the following experiment: dissolving compound **2 b** in THF, and subsequent removal of the solvent under vacuum, and treatment of the residue at 110 °C under high vacuum for 6 h gave a material whose elemental analysis indicated the reformation of **2 b**; however, the ^1^H NMR spectrum of such reformed material revealed a considerably changed **A**/**B**/**C** integral ratio, when redissolved in [D_8_]THF (Figures S10 and S11). For further comparison, homoleptic gallium methylene undergoes a reversible [Ga_8_(*μ*‐CH_2_)_12_]/[Ga_6_(*μ*‐CH_2_)_9_](Do)_x_ oligomer switch in donor (Do) solvents including THF.^[22]^

**Scheme 3:**
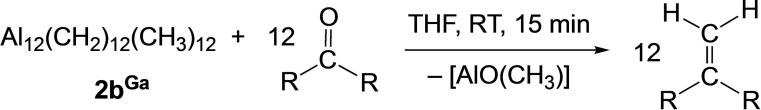
Methylidene‐transfer reactivity of [(CH_3_)_12_Al_12_(μ_3_‐CH_2_)_12_] (**2 b**) with 9‐fluorenone, benzophenone or acetone.

Compound **2 b** promotes carbonyl methylenation as efficiently as the Tebbe reagent or related rare‐earth‐metal variants.^[11]^ Treatment of compound **2 b** with 12 equiv. 9‐fluorenone, benzophenone or acetone at ambient temperatures in [D_8_]THF resulted in the complete consumption of the AlCH_2_ moieties and conversion to 9‐methylene‐fluorene, 1,1‐diphenylethylene and isobutene, respectively, within 15 minutes (Scheme 3, Figures S23–S25). Unsurprisingly, aluminium methylene **2 b** converts the carbonylic substrates considerably faster than gallium methylene [Ga_8_(CH_2_)_12_] (RT, 5 d), featuring an increasingly covalent Ga−C bond.^[22]^ It should be noted that, as indicated by ^1^H NMR spectroscopy, no carbonyl alkylation was observed, leaving putative methylaluminoxane (MAO) as a coproduct.

## Conclusion

After more than 50 years since its first appearance, the structure of methylaluminomethylene could be elucidated. Crucially, a transmetalation reaction involving gallium methylene [Ga_8_(CH_2_)_12_] and AlMe_3_ has proven expedient. The crystal structure of [(CH_3_)_12_Al_12_(*μ*_3_‐CH_2_)_12_] exhibits {Al(CH_2_)_4_} tetrahedra as an organometallic variant of the ubiquitous {AlO_4_} tetrahedra. The aluminium‐methylene moieties efficiently engage in carbonyl olefination reactions.

The synthesis procedures are described in the Supporting Information.

Deposition Numbers 2157627 (for **1**), 2157626 (for **2 a**), and 2157628 (for **2 b**) contain the supplementary crystallographic data for this paper. These data are provided free of charge by the joint Cambridge Crystallographic Data Centre and Fachinformationszentrum Karlsruhe Access Structures service.

## Conflict of interest

The authors declare no conflict of interests.

1

## Supporting information

As a service to our authors and readers, this journal provides supporting information supplied by the authors. Such materials are peer reviewed and may be re‐organized for online delivery, but are not copy‐edited or typeset. Technical support issues arising from supporting information (other than missing files) should be addressed to the authors.

Supporting Information

## Data Availability

The data that support the findings of this study are available in the supplementary material of this article.
