# Supplementary material for: [(CH3)Al(CH2)]12: Methylaluminomethylene (MAM‐12)
Source: Chemistry. 2022 Jul 7;28(48):e202200823. doi: 10.1002/chem.202200823 (PMC9544092; doi:10.1002/chem.202200823)
Supplement: Supplementary file 1 — Supporting Information [file CHEM-28-0-s001.pdf]

Chemistry–A European Journal

Supporting Information

$[(CH_3)Al(CH_2)]_{12}$: Methylaluminomethylene (MAM-12)

Georgios Spiridopoulos, Markus Kramer, Felix Kracht, Cäcilia Maichle-Mössmer, and Reiner Anwander*

Table of Contents

Experimental Section S3
NMR and EPR Spectra S5
X-Ray Crystallography S22
IR Spectra S24
References S24

Experimental Section

General Considerations. All operations were performed under rigorous exclusion of air and water by using standard Schlenk, high-vacuum, and glovebox techniques (MBraun 200B; <0.1ppm O_2, <0.1 ppm H_2O). Solvents were purified by using Grubbs-type columns (MBraun SPS, solvent purification system) and stored inside a glovebox. [D$_8$]THF was obtained from Sigma-Aldrich, stirred over NaK and distilled. [D$_6$]benzene was obtained from Sigma-Aldrich stirred over NaK and filtered at ambient temperature. $Cp_2Ti(\mu\text{-}CH_2)(\mu\text{-}Cl)AlMe_2$,[1] $Ga_8(CH_2)_{12}$,[2] and $Cp^*_2Lu(AlMe_4)$[2] were synthesized according to literature procedures. $AlMe_3$, 9-fluorenone, and benzophenone were purchased from Sigma-Aldrich and used as received. Acetone was purchased from Honeywell and a small amount was stored in the glovebox over molecular sieve. If not otherwise stated, the NMR spectra were recorded by using J.Young-valved NMR tubes on a Bruker AVII+400 spectrometer (^1H, 400.13 MHz; ^{13}C, 100.61 MHz) and a Bruker AVII+500 spectrometer (^1H, 500.13 MHz; ^{13}C, 125.76 MHz) at 26 °C. NMR chemical shifts are referenced to internal solvent resonances and reported in parts per million relative to tetramethylsilane (TMS). All pulse gradient spin echo-NMR measurements were performed on an Avance III HD spectrometer (Bruker) operating at 700.29 MHz for ^1H, using a TCI prodigy cryoprobe head equipped with a z-gradient unit. The gradient was calibrated using "doped water" (1% H_2O in D_2O with traces of $CuSO_4$) assuming a diffusion coefficient of 1.91·10-5 cm^2 s^{-1} for HDO. The diffusion measurements used a modified bipolar gradient pulse pair-stimulated echo sequence incorporating a longitudinal eddy current delay (BPP-LED). The gradient pulse length (δ) and the diffusion time (Δ) were kept at fixed values while gradually increasing the gradient strength. Typical values for δ and Δ were 1,6 and 75 ms, respectively. A longitudinal eddy cur-rent delay (Te) of 5 ms was used. Sine-shaped gradient pulses were linearly varied between 1 and 52 G cm^{-1} (2 to 98%) in 32 steps and at each step 16 scans were acquired. Four measurements per sample were performed at a constant sample temperature of 298 ± 0.1 K (Bruker Variable Temperature Unit BCU II). The data were analyzed with the T_1/T_2 relaxation module of Topspin 4.1.3. The signal areas were plotted against the gradient strength and the best fit was calculated using the Stejskal-Tanner equation

$$I_g = I_0 \cdot \exp [-4\pi^2\gamma^2\delta^2G^2(\Delta\text{-}\ \delta/3)]$$

(with D being the diffusion coefficient in cm^2 s^{-1}, γ the gyromagnetic ratio in Hz/G, G the gradient strength in G cm^{-1}, δ the gradient length in ms, Δ the interval between gradient pulses (diffusion time) in ms, I_g the signal area, and I_0 the signal intensity at G = 0%). Mean values for each sample are reported. EPR spectra were measured on a continuous wave X-band Bruker ESP 300E using 5 mm O.D. Wilmad quartz (CFQ) EPR tubes. Spectra are referenced to the Bruker strong pitched standard g_{iso} = 2.0088. Elemental analyses were performed on an Elementar Vario Micro Cube. IR spectra were recorded on a Thermo Fisher Scientific NICOLET 6700 FTIR spectrometer using a DRIFT chamber with dry KBr/sample mixture and KBr windows. The DRIFT data were converted using the Kubelka-Munk refinement. Additionally, IR spectra were measured on a Bruker Vertex 70 using CsI plates with Nujol.

[$Cp^*_4Lu_2Al_{10}(CH_2)_{12}(CH_3)_8$] (1): A solution of $Cp^*_2LuAlMe_4$ (21 mg, 0.039 mmol) in toluene-d_8 (0.5 mL) was added to a stirred solution of $AlMe_3$ (11.3 mg, 0.157 mmol) in toluene-d_8 (0.2 mL). The reaction mixture was allowed to stir for 15 min at ambient temperature. Afterwards the reaction mixture was transferred to a J.Young-valved NMR tube and heated at 130 °C for 500 h. Colorless single crystals were obtained at 130 °C from the reaction mixture. ^1H NMR (400.11 MHz, THF-d_8, 26 °C): δ 1.87 (s, 60 H, Cp*Me), –0.96 (s, Al–CH_2 / Al–CH_3), –0.99 (s, Al–CH_2 / Al–CH_3), –1.16 (s, Al–CH_2 / Al–CH_3) ppm. Due to the small available amount of compound **1**, any further analysis could not be performed. Repeated attempts to obtain additional crystalline compound **1**, both under identical and similar reaction conditions, were unsuccessful. The similar reaction conditions included heating of $Cp^*_2LuAlMe_4/AlMe_3$ mixtures (ratios: 1:4, 1:6, 1:8) in toluene-d_8 (0.5 mL) at 130 °C for 500±20 h.

2^{Tebbe} (Tebbe route): a) In a glovebox, $Cp_2Ti(\mu\text{-}Cl)(\mu\text{-}CH_2)(AlMe_2)$ (26 mg, 0.092 mmol) was stirred in neat $AlMe_3$ (659.7 mg, 9.15 mmol) at ambient temperature. While stirring for 170 h methane evolution occurred and a red solid precipitated. The precipitate was subjected to Soxhlet extraction (benzene, 3 d), washed with benzene (3 x 5 mL), and evaporated to dryness in vacuo to yield **2^{Tebbe}** as a red solid (350 mg, 0.520 mmol, 68%). ^1H NMR (400.11 MHz, THF-d_8, 26 °C, cluster fragmentation):): δ –0.96 (s, 24H, Al–CH_3), –0.99 (s, 22H, Al–CH_3), –1.03 (s, 18H, Al–CH_3), –1.81 (s, 24H, Al–CH_2) ppm. $^{13}C\{^1H\}$ NMR (100.6 MHz, THF-d_8, 26 °C): δ –5.1 (Al–CH_2), –6.9 (Al–CH_3), –7.9 (Al–CH_3), –9.4 (Al–CH_3) ppm. Elemental analysis [%]: Calcd. C 42.86, H 8.99; found C 41.54, H 7.66. ICP-OES : Ti 0.33%. IR (Nujol) [cm^{-1}]: v = 2952, 2923, 2854, 1456, 1377, 1199 (w), 687 (w), 611 (w), 525 (w).

b) In a glovebox, Cp_2TiCl_2 (20 mg, 0.08 mmol) was stirred in neat $AlMe_3$ (579 mg, 8.03 mmol) at ambient temperature. While stirring for 7 d methane evolution occurred and a red solid precipitated. The precipitate was washed with benzene (3 x 5 mL) and evaporated to dryness in vacuo to yield **2^{Tebbe}** as a reddish solid (329 mg, 0.489 mmol, 73%) with characterization data comparing to the material isolated via procedure a).

[$(CH_3)_{12}Al_{12}(CH_2)_{12}$] (2a/b) (gallium-methylene route): a) A J.Young-valved NMR tube was charged with (51 mg, 0.07 mmol) of $Ga_8(CH_2)_{12}$. Then, a solution of $AlMe_3$ (60.7 mg, 0.84 mmol, 12 equivalents) in 0.5 mL C_6D_6 was added. The J. Young NMR tube was removed from the glovebox and heated in an oil bath at 70 °C overnight. Colorless single crystals of **2a** were obtained at 70 °C from the reaction mixture. Due to the contamination with gallium, no further analysis was performed.

(b) In a glovebox, a pale yellow suspension of $Ga_8(CH_2)_{12}$ (100 mg, 0.14 mmol) in *n*-hexane (3 mL) and excess $AlMe_3$ (238.3 mg, 3.31 mmol, 24 equivalents) in *n*-hexane (2 mL) were stirred together at ambient temperature. After stirring for 16 h a colorless solid precipitated. The precipitate was washed with benzene (3 x 5 mL) and dried in vacuo to yield **2b** as white solid (80 mg, 0.12 mmol, 85%). ^{1}H NMR (400.11 MHz, THF-d_8, 26 °C): δ –0.97 (s, 22H, Al–CH_3), –0.99 (s, 18H, Al–CH_3), –1.04 (s, 24H, Al–CH_3), –1.81 (s, 24H, Al–CH_2) ppm. Cluster fragmentation was revealed by DOSY measurements (see Figure S16). ^{13}C{^{1}H NMR (100.6 MHz, THF-d_8, 26 °C): δ –4.8 (Al–CH_2), –6.6 (Al–CH_3), –7.6 (Al–CH_3), –9.2 (Al–CH_3) ppm. ^{27}Al NMR (130.32 MHz, THF-d_8, 26 °C): δ –180.7 (very broad) ppm. Elemental analysis [%] $Al_{12}(CH_2)_{12}(CH_3)_{12}$ (672.52 g mol^{-1}): Calcd. C 42.86, H 8.99; found C 43.37, H 8.51. ICP-OES: Ga 0.66%, Al 34.21%. DRIFT (KBr): ν 2926 (w), 2887 (w), 1324 (vw), 1249 (vw), 1198 (s), 824 (s), 696 (s), 662 (s), 663 (s), 649 (s), 613 (s), 547 (s) cm^{-1}. IR (Nujol) [cm^{-1}]: ν = 2953 (Nujol), 2924 (Nujol), 2854 (Nujol), 1461 (Nujol), 1377 (Nujol), 1199 (w), 817 (w), 721 (w), 688 (w), 611 (w), 521 (w). Compound **2b** (10 mg) was dissolved in 0.5 mL THF and stirred for 5 min. After removing the solvent under vacuum, the residue was heated to 110 °C at a high vacuum unit for 6 h, without any color change. Elemental analysis of the residue indicated the reformation of **2b**: calcd. C 42.86, H 8.99; found C 43.48, H 8.44 (see also Figure S11). Single crystals of **2b** were obtained by heating a suspension of $Ga_8(CH_2)_{12}$ (50.4 mg, 0.07 mmol) and $AlMe_3$ (117.6 mg, 1.66 mmol, 24 equivalents) in benzene (1 mL) in a J.-Young-valved NMR tube to 70 °C overnight (16 h). The crystals of **2b** formed at 70 °C, attaching to the glass wall at the liquid/gas interface.

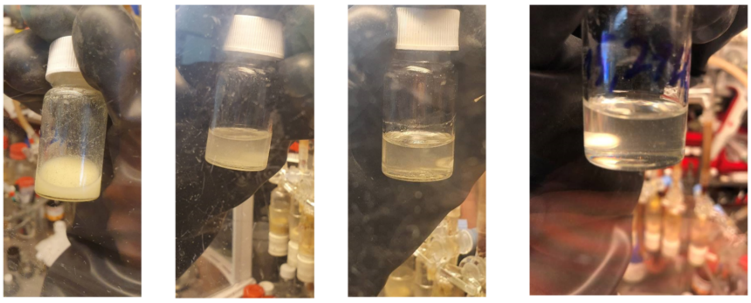

5 min 10 min 15 min 30 min

Figure S1. Gradual dissolution of gallium methylene $Ga_8(CH_2)_{12}$ (suspension in *n*-hexane) upon addition of excess of $AlMe_3$ (solution in *n*-hexane). The reaction progress, involving the formation of methylaluminomethylene MAM as a white precipitate, is not shown.

General procedure for carbonyl olefination experiments. A J.-Young-valved NMR tube was charged with 10 mg of [$(CH_3)_{12}Al_{12}(CH_2)_{12}$] (**2b**). Subsequently, 12 equivalents of the respective carbonylic compound dissolved in 0.4 mL THF-d_8 was added and after 15 min a ^{1}H NMR spectrum was recorded (see Figures S24-S26).

NMR and EPR Spectra

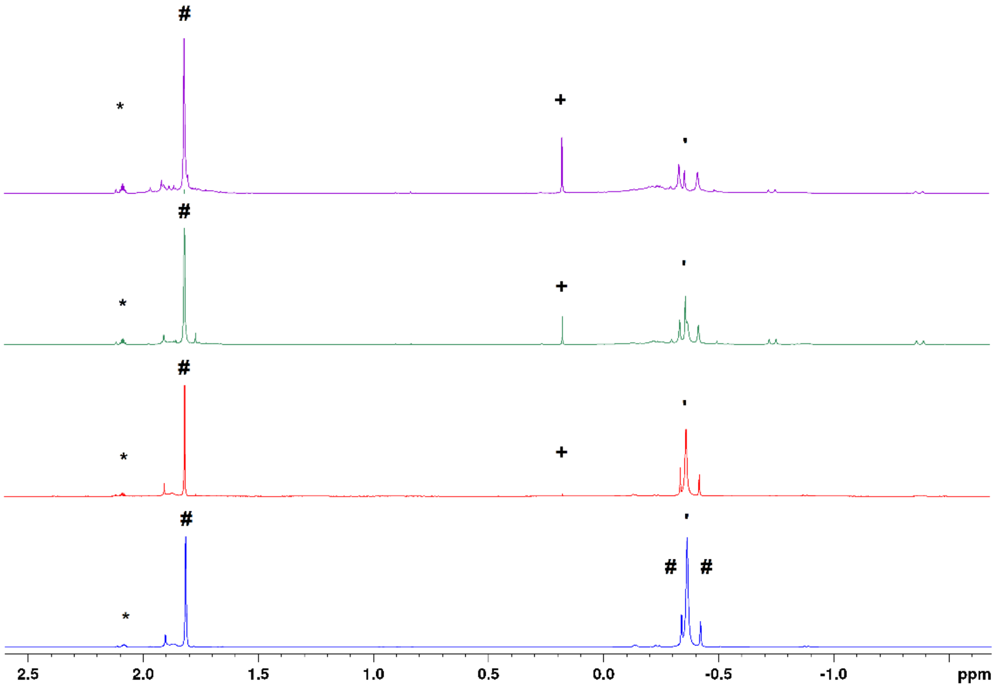

Figure S2. ^{1}H NMR spectra (400.11 MHz) of the reaction of [Cp*$_2$Lu(AlMe$_4$)] (#) with AlMe$_3$ (‘) toward complex **1** in toluene-*d*$_8$ (*). From bottom to top : 20 min, RT (blue), 40 h, 130 °C (red), 240 h, 130 °C (green) and 500 h at 130 °C (purple). Decrease of the AlMe$_3$ signal (‘) and simultaneous appearance of the signal of methane (+) and signals in the (high field) range assignable to [Al–CH$_2$] moieties are observed.

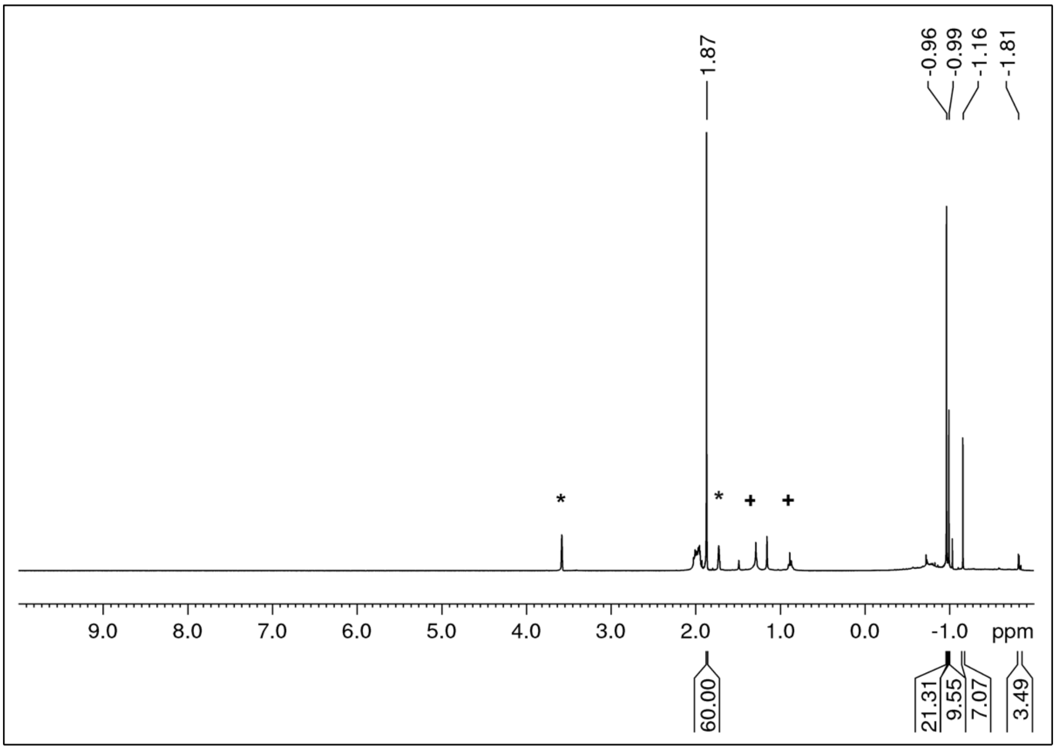

Figure S3a. ^{1}H NMR spectrum (400.11 MHz) of [Cp*$_4$Lu$_2$Al$_{10}$(CH$_2$)$_{12}$(CH$_3$)$_8$] (**1**) in THF-*d*$_8$ (*). Assumed fragmentation of **1** is indicated by the appearance of a high-field AlCH$_3$/AlCH$_2$ signal pattern similar to that of a solution of **2b** in THF. The signal at 1.87 ppm is assigned to the remaining [Cp*$_2$Lu] fragment. (+) *n*-hexane, from washings of compound **1**.

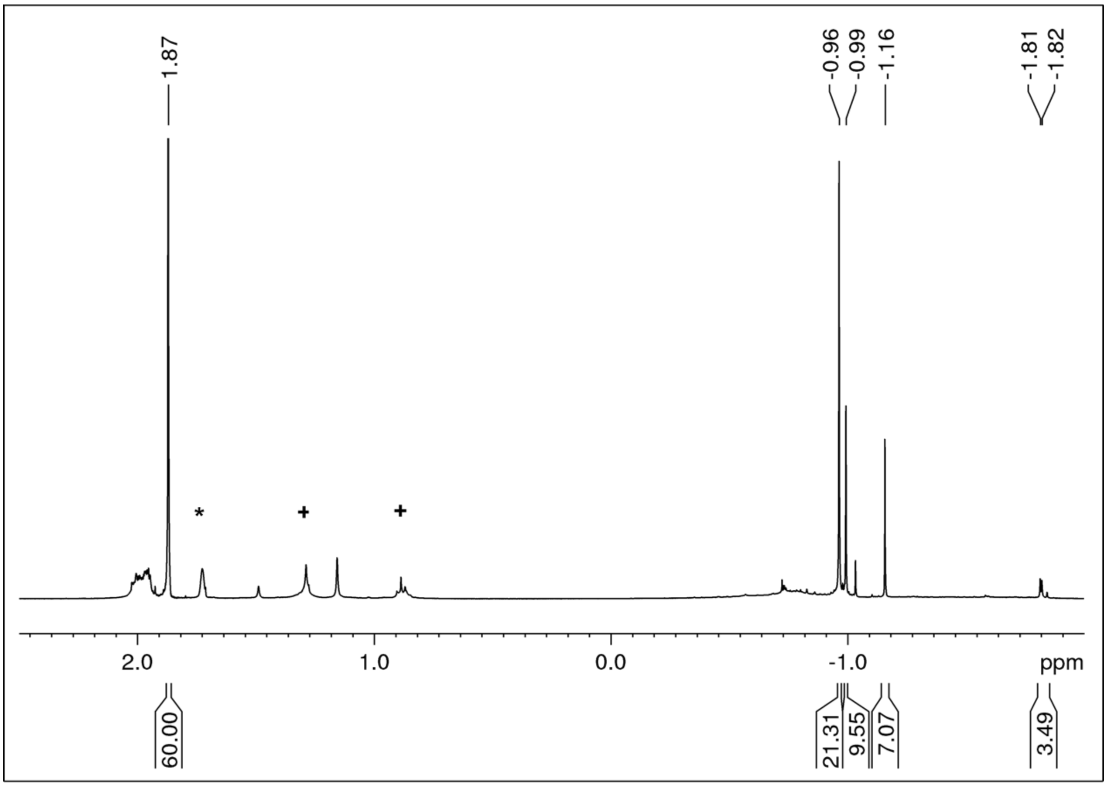

Figure S3b. Section of the ^{1}H NMR spectrum (400.11 MHz) of $[Cp^*_4Lu_2Al_{10}(CH_2)_{12}(CH_3)_8]$ (**1**) in THF-d_8 (*). Assumed fragmentation of **1** is indicated by the appearance of a high-field $AlCH_3/AlCH_2$ signal pattern similar to a solution of **2b** in THF. The signal at 1.87 ppm is assigned to the remaining $[Cp^*_2Lu]$ fragment.

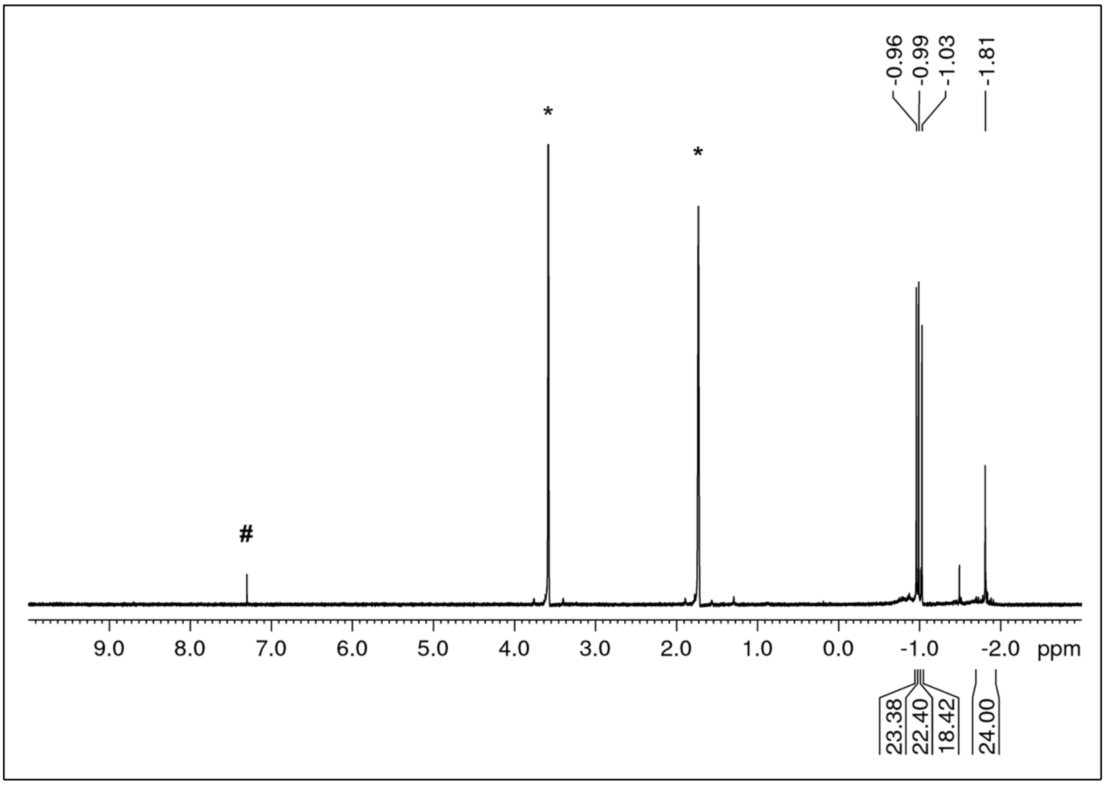

Figure S4. ^{1}H NMR spectrum (400.11 MHz) of $\mathbf{2}^{\mathbf{Tebbe}}$ obtained via the Tebbe route in THF-d_8 (*).

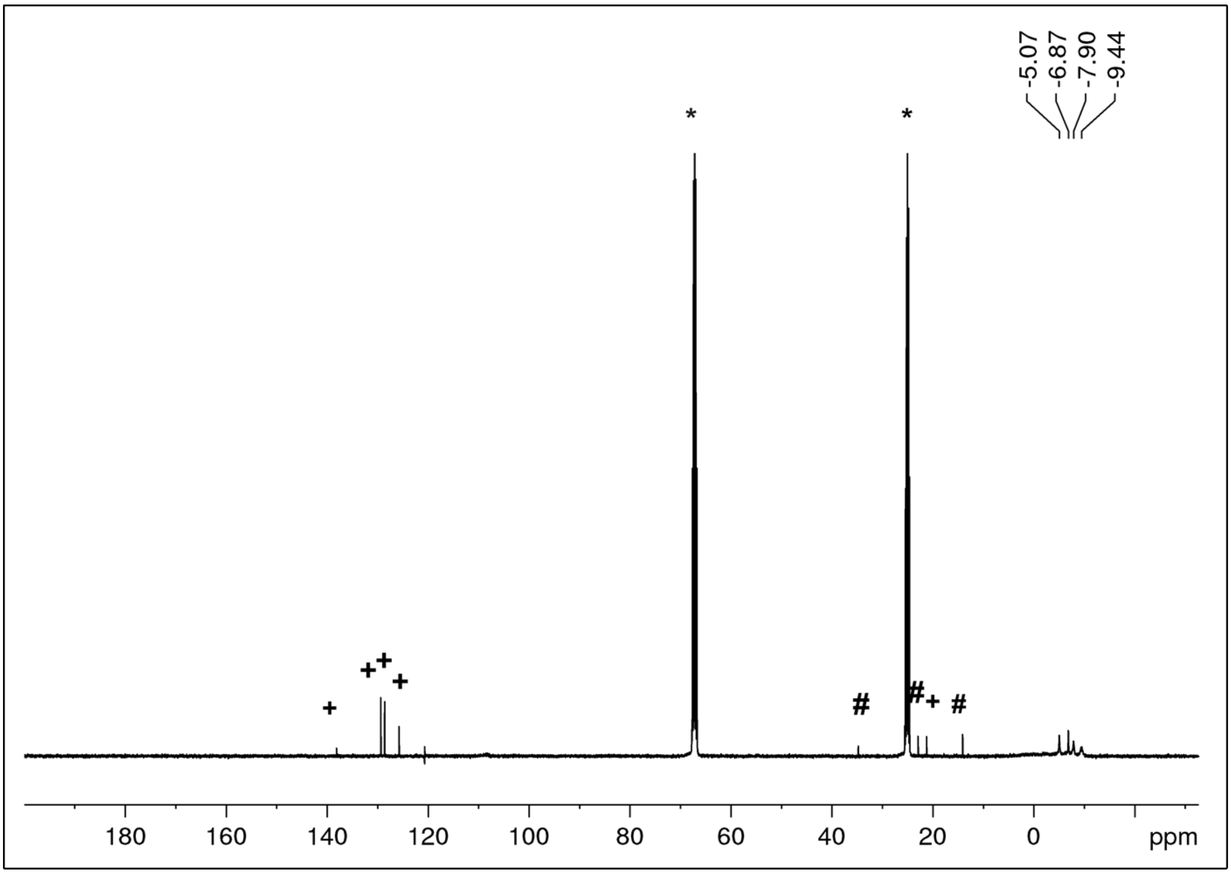

Figure S5. ^{13}C NMR spectrum (100.6 MHz) of $\mathbf{2^{Tebbe}}$ in THF-d_8 (*); *n*-pentane (#) and toluene (+) from washings.

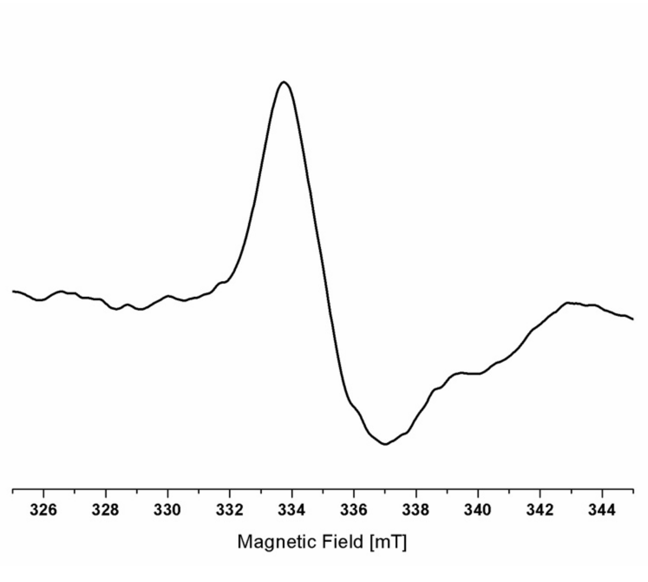

Figure S6. X-band cw-EPR spectrum of $\mathbf{2^{Tebbe}}$ (neat).

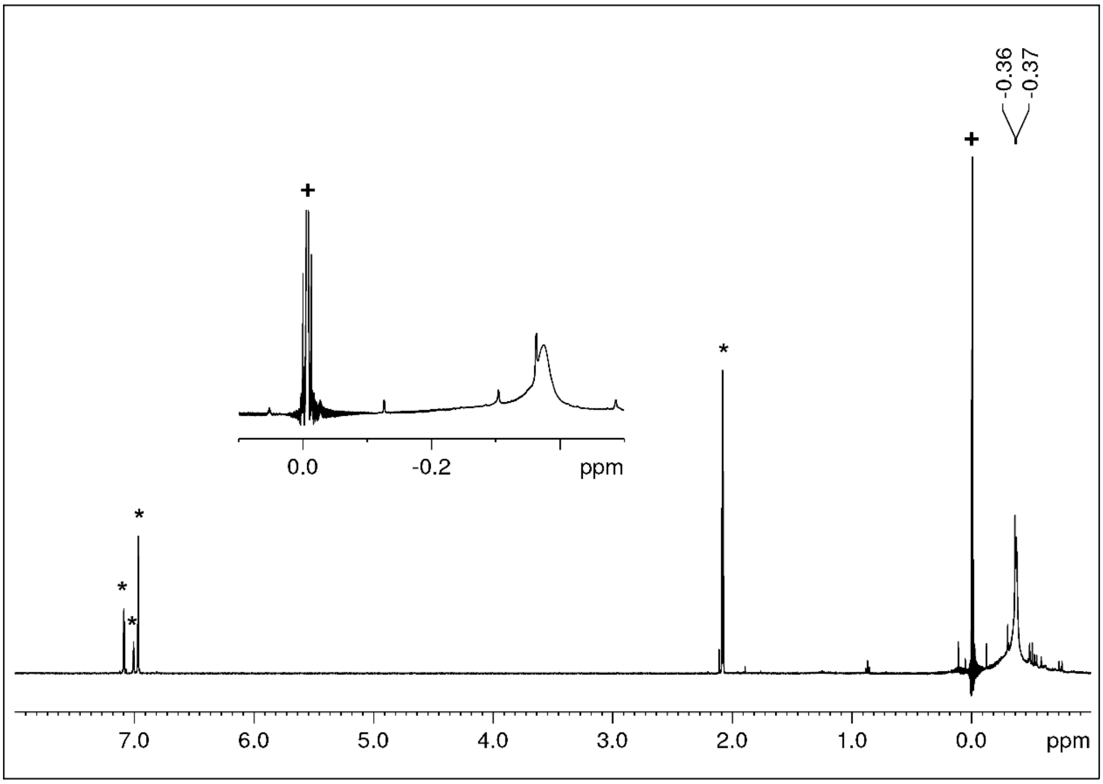

Figure S7. ^{1}H NMR spectrum (500.13 MHz) of "[$(CH_3)_{12}Al_{12}(CH_2)_{12}$] (**2b**)" in toluene-$d_8$ (*) at 26 °C. Signal from TMS is marked with +.

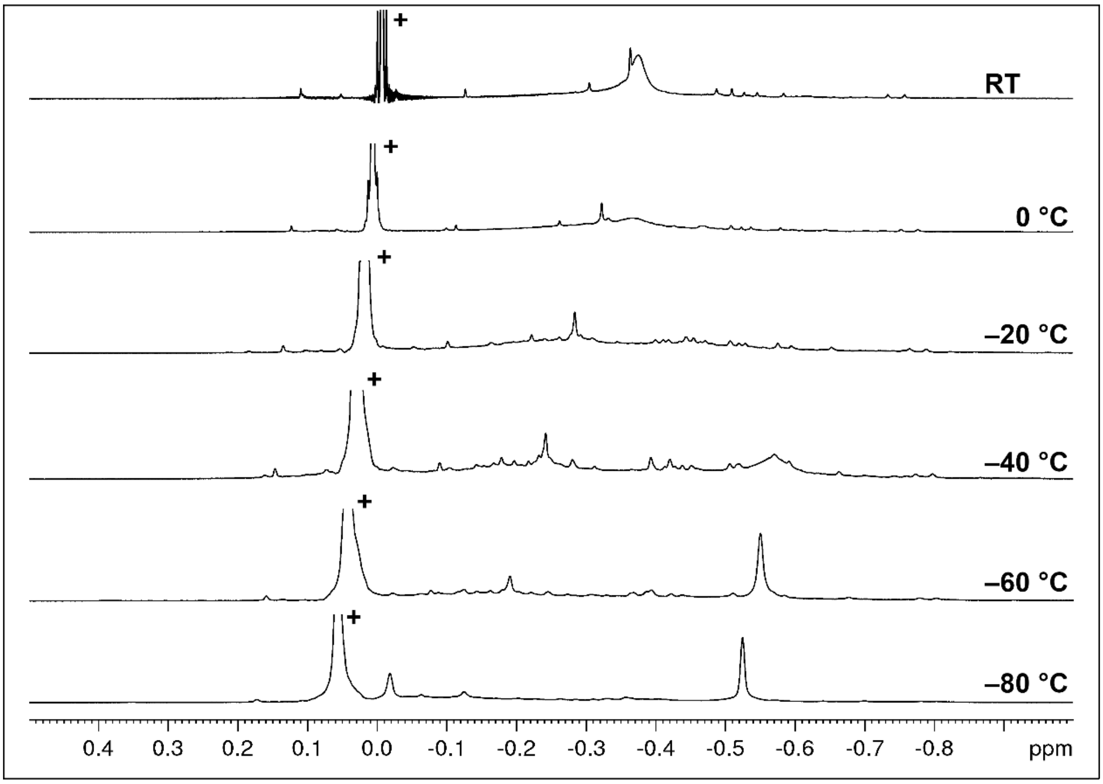

Figure S8. Variable temperature ^{1}H NMR spectra (500.13 MHz) of "[$(CH_3)_{12}Al_{12}(CH_2)_{12}$] (**2b**)" in toluene-$d_8$. Signal from TMS is marked with +.

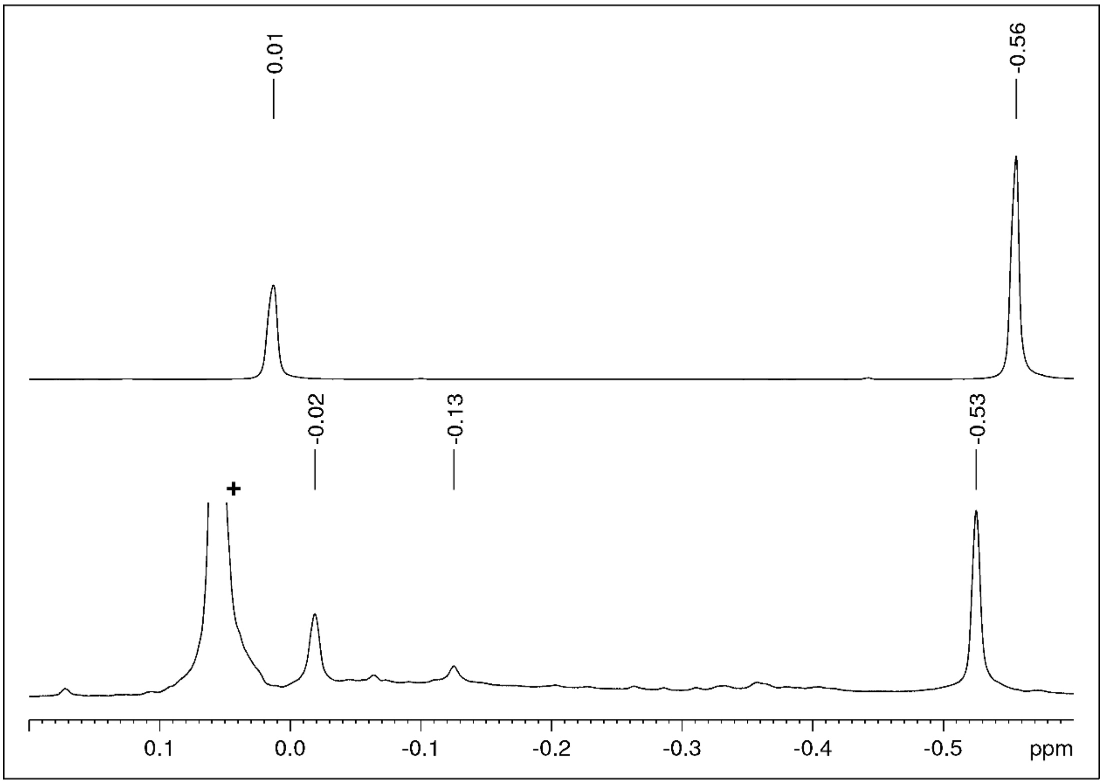

Figure S9. Comparison of the ^{1}H NMR spectra (500.13 MHz) of $AlMe_3$ (top) and "$[(CH_3)_{12}Al_{12}(CH_2)_{12}]$ (**2b**)" (bottom) in toluene-d_8 at –80 °C. Signal from TMS is marked with +.

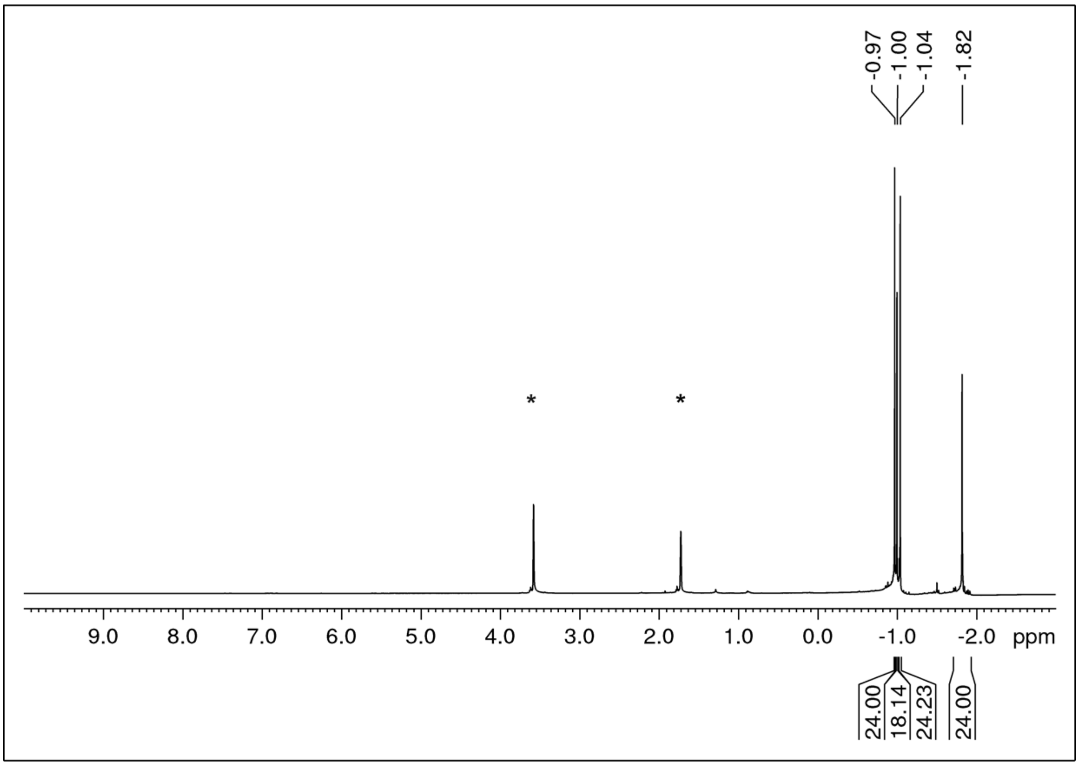

Figure S10. ^{1}H NMR spectrum of (400.11 MHz) $[(CH_3)_{12}Al_{12}(CH_2)_{12}]$ (**2b**) obtained via the gallium methylene route (24 equiv.) in THF-d_8 (*).

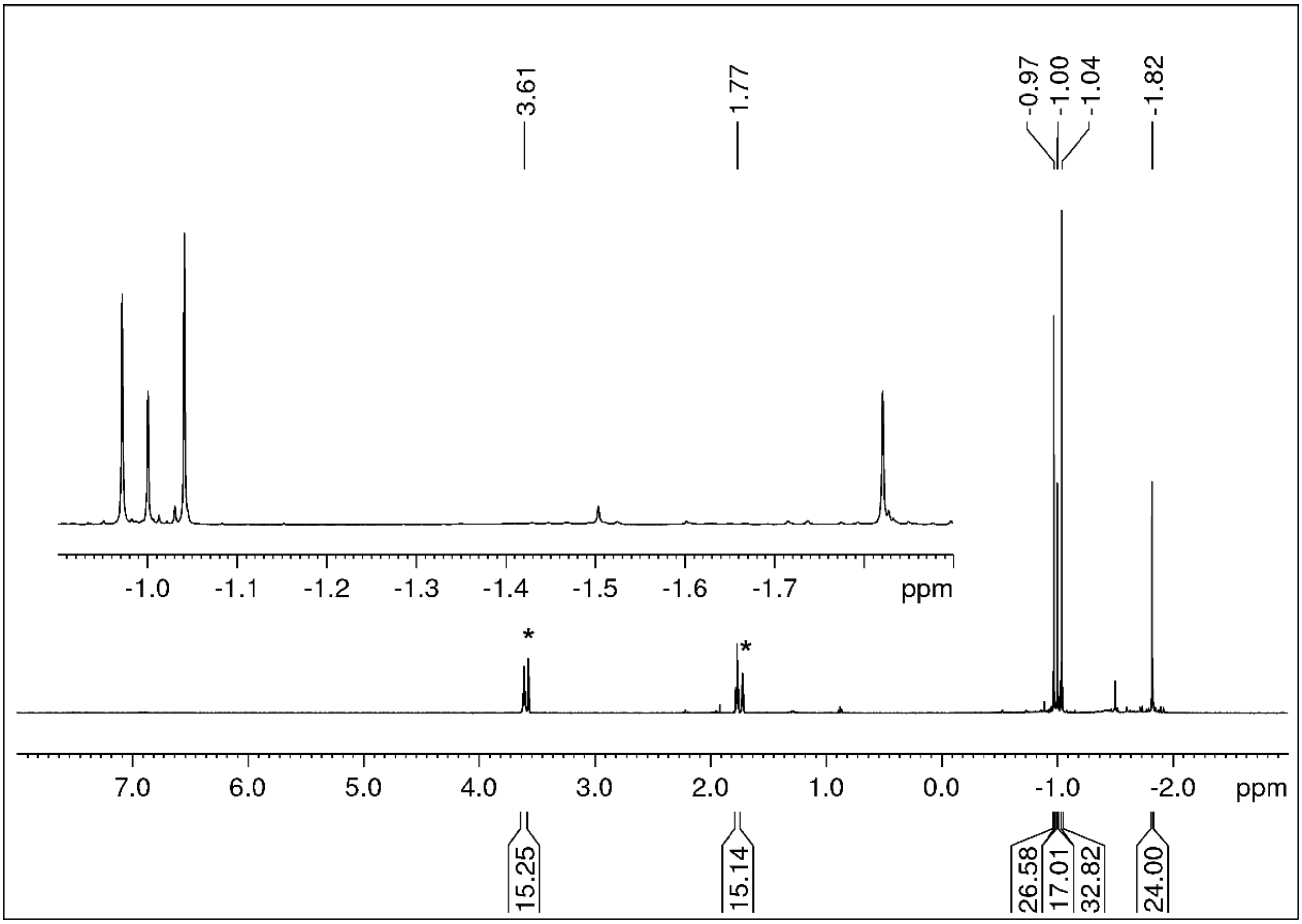

Figure S11. ^{1}H NMR spectrum (500.13 MHz) of a sample of $[(CH_3)_{12}Al_{12}(CH_2)_{12}]$ (**2b**, obtained via the gallium methylene route (24 equiv.)), after dissolving in THF for 5 min, removing the solvent under vacuum, heating the residue to 110 °C under high vacuum for 6 h, and redissolving it in THF-d_8 (*).

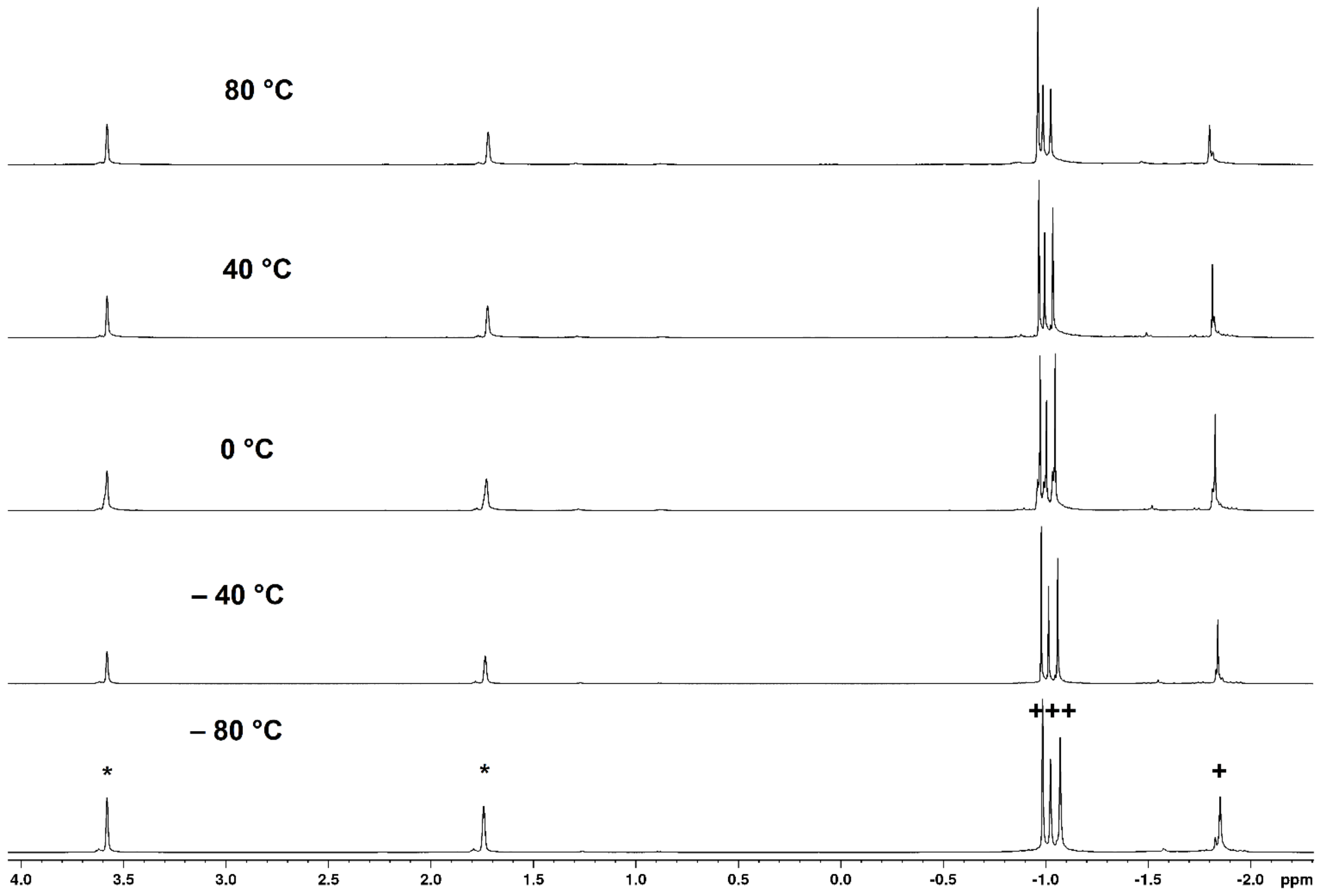

Figure S12. Variable-temperature ^{1}H NMR spectra (500.13 MHz) of complex [$(CH_3)_{12}Al_{12}(CH_2)_{12}$] (**2b**) obtained via the gallium methylene route (24 equiv.) in THF-d_8 (*)..

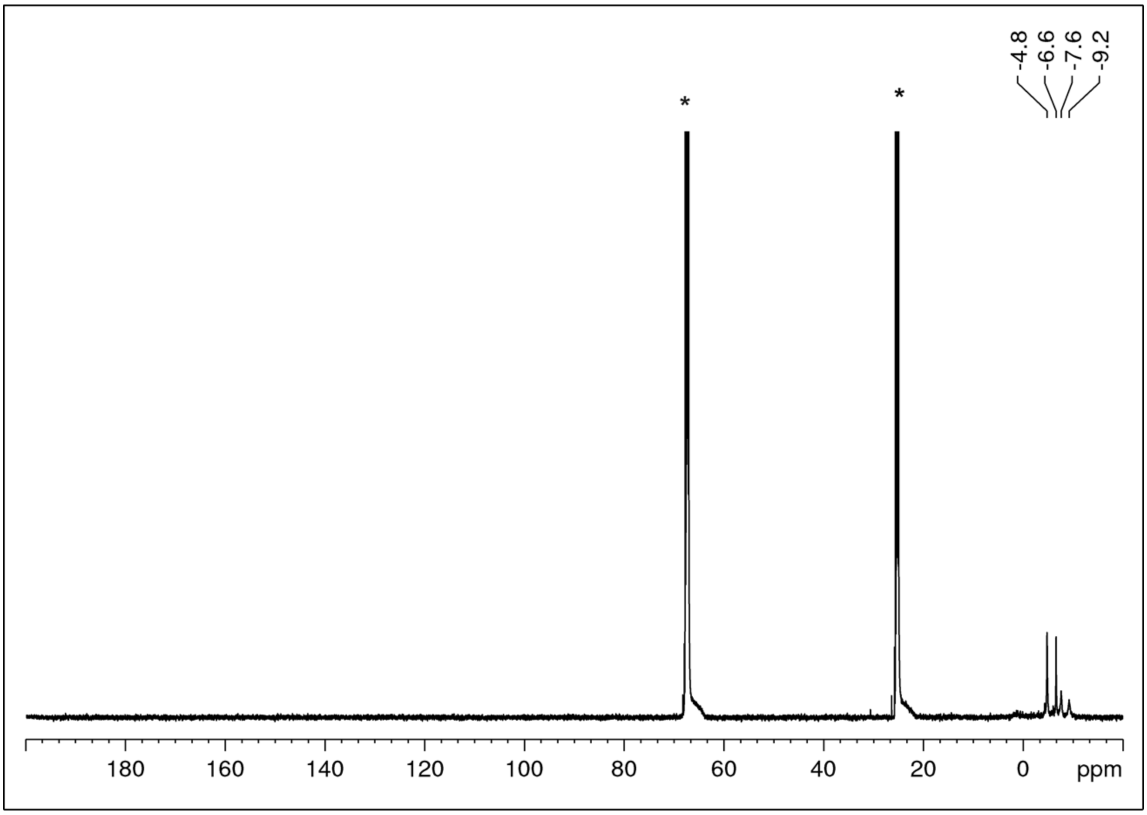

Figure S13. ^{13}C NMR spectrum (100.6 MHz) of $[(CH_3)_{12}Al_{12}(CH_2)_{12}]$ (**2b**) obtained via the gallium methylene route (24 equiv.) in THF-d_8 (*).

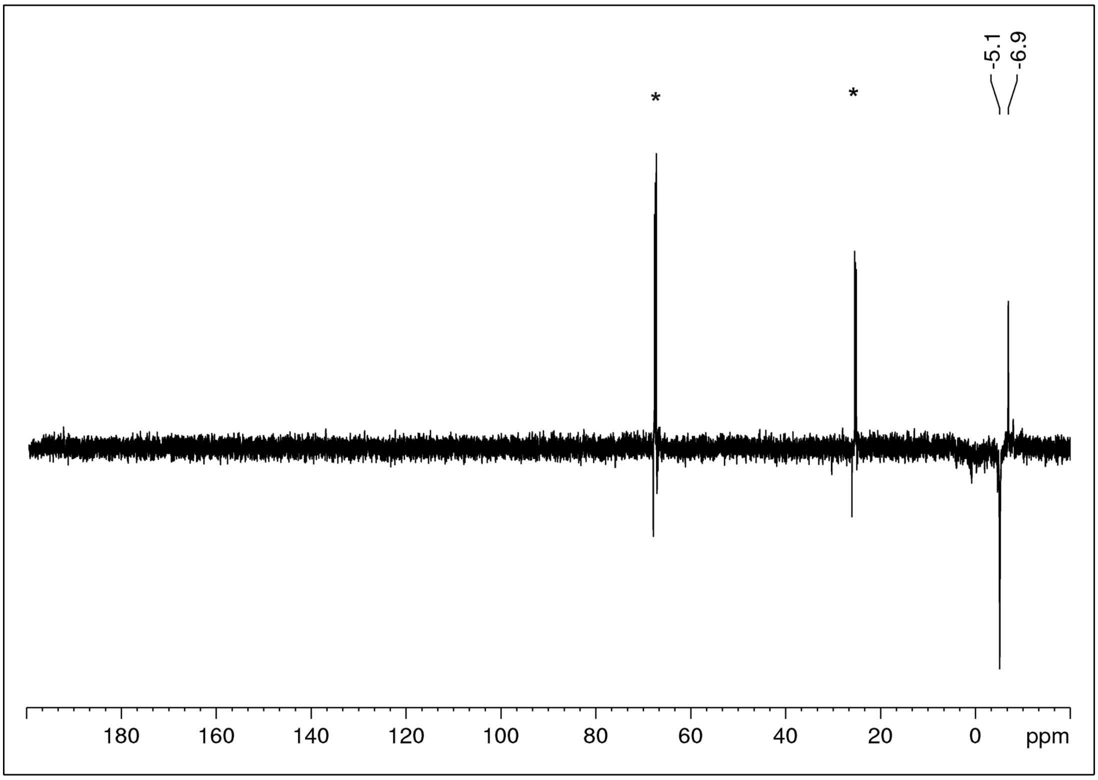

Figure S14. ^{13}C-DEPT135 NMR spectrum of $[(CH_3)_{12}Al_{12}(CH_2)_{12}]$ (**2b**) obtained via the gallium methylene route (24 equiv.) in THF-d_8 (*).

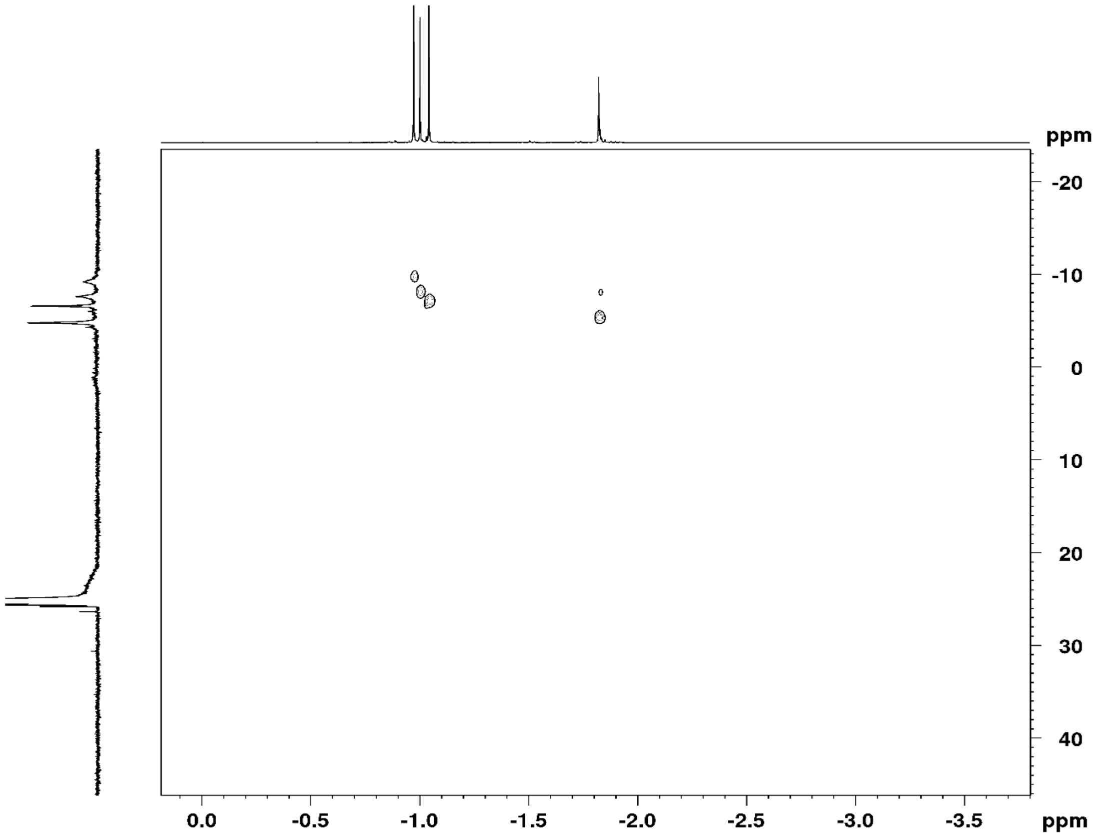

Figure S15. ^{1}H–^{13}C HSQC NMR spectrum of $[(CH_3)_{12}Al_{12}(CH_2)_{12}]$ (**2b**) obtained via the gallium methylene route (24 equiv.) in THF-d_8 (*).

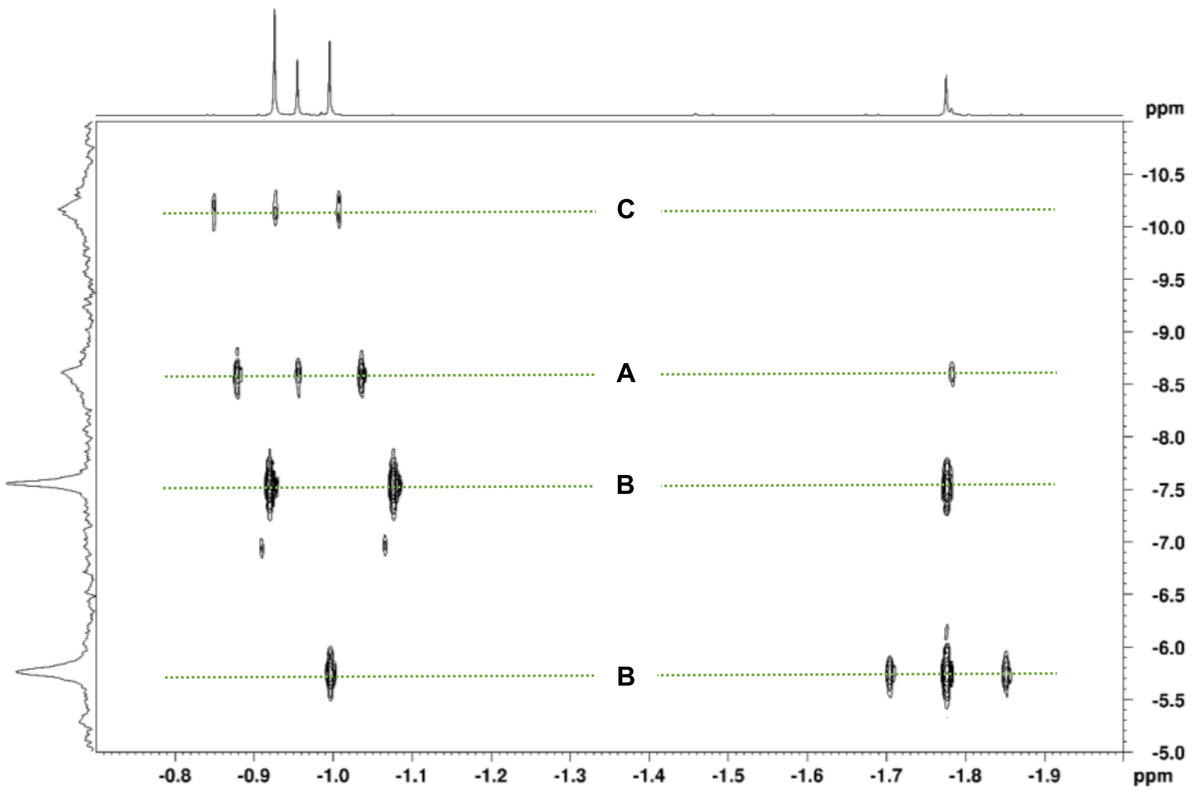

Figure S16. ^{1}H–^{13}C HMBC NMR spectrum of of [$(CH_3)_{12}Al_{12}(CH_2)_{12}$] (**2b**) obtained via the gallium methylene route (24 equiv.) in THF-d_8 (*). The denomination of the cross peaks corresponds to the structure denomination shown in Figure S20.

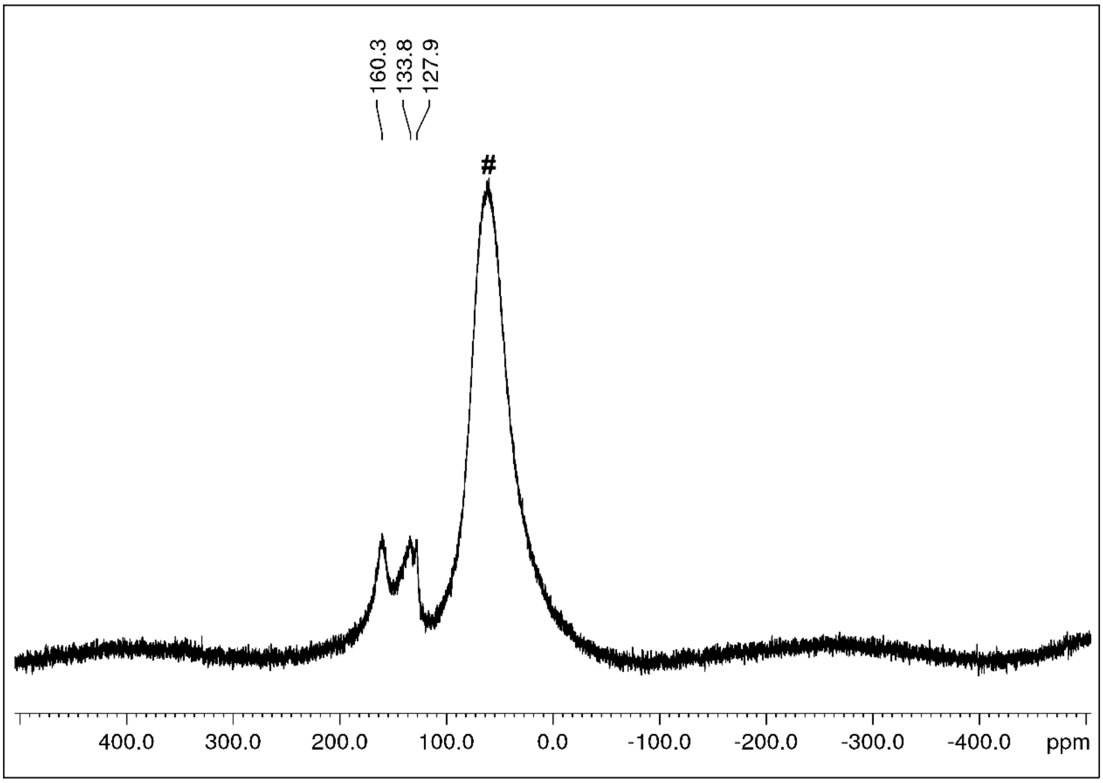

Figure S17. ^{27}Al NMR spectrum (130.32 MHz) of $[(CH_3)_{12}Al_{12}(CH_2)_{12}]$ (**2b**) in toluene-d_8. Signal from probe head is marked with #.

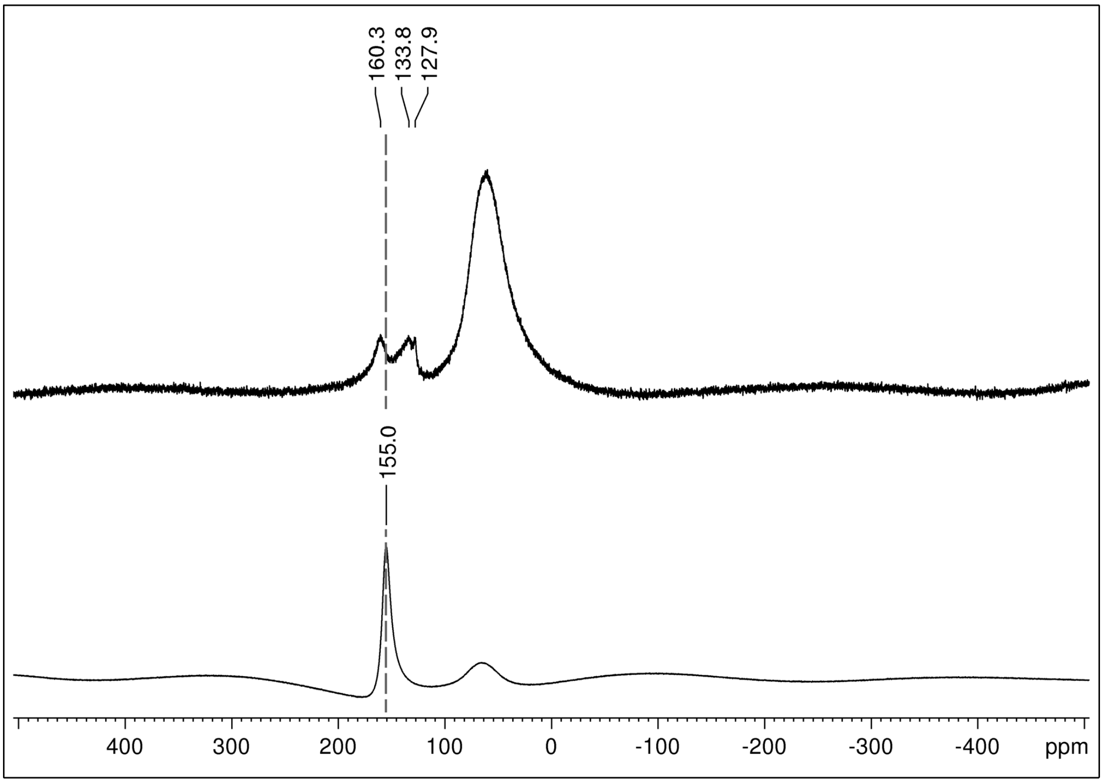

Figure S18. Comparison of the ^{27}Al NMR spectra (130.32 MHz) of $[(CH_3)_{12}Al_{12}(CH_2)_{12}]$ (**2b**, top) and $AlMe_3$ (bottom) both in toluene-d_8.

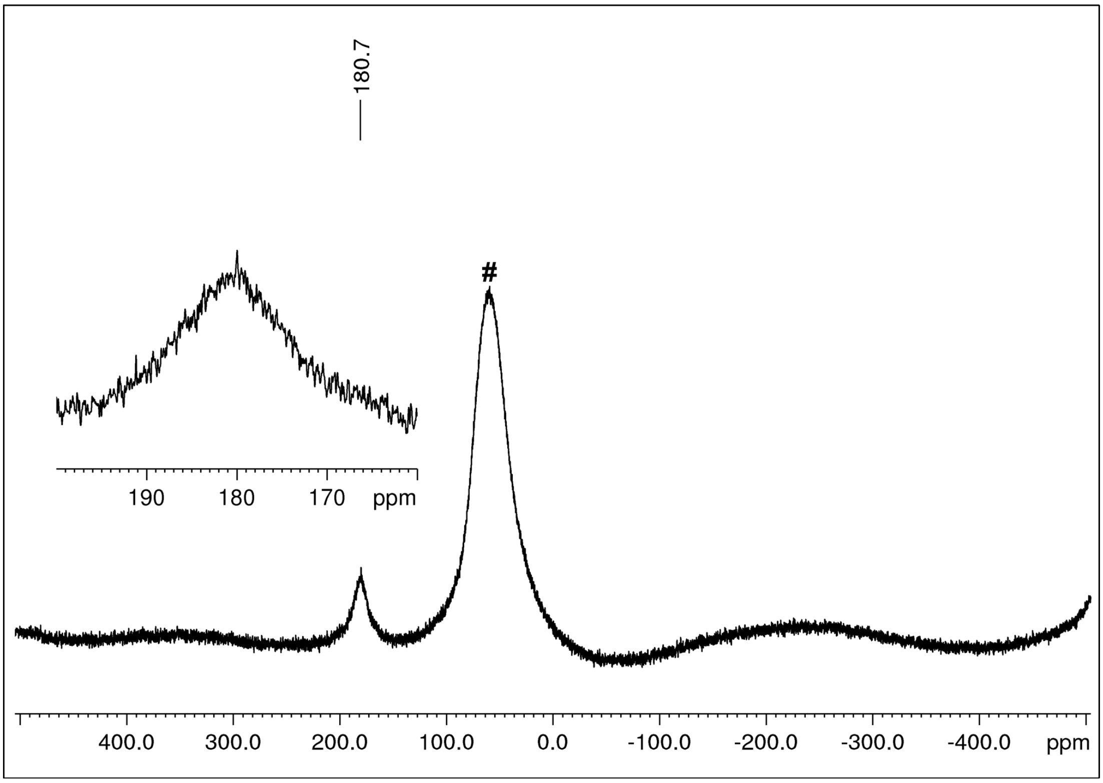

Figure S19. ^{27}Al NMR spectrum (130.32 MHz) of [$(CH_3)_{12}Al_{12}(CH_2)_{12}$] (**2b**) in THF-$d_8$. Signal from probe head is marked with #.

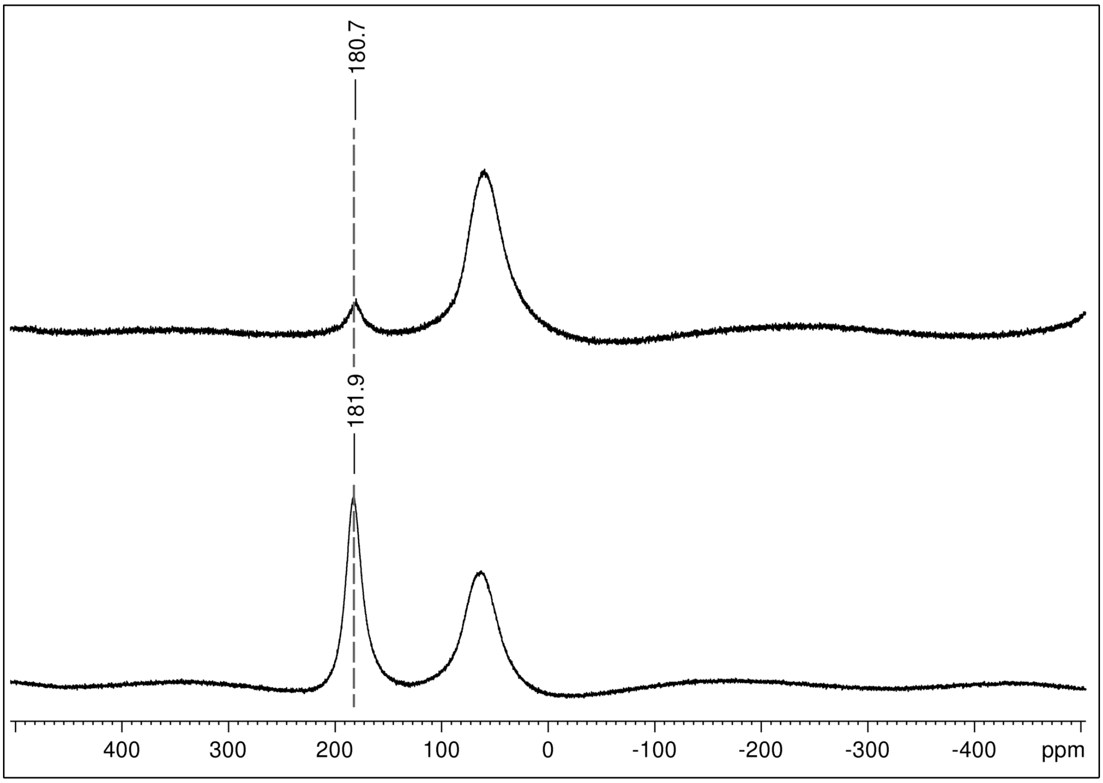

Figure S20. Comparison of the ^{27}Al NMR spectra (130.32 MHz) of [$(CH_3)_{12}Al_{12}(CH_2)_{12}$] (**2b**, top) in THF-$d_8$ and $AlMe_3$(THF) (bottom) in toluene-d_8.

PGSE/DOSY Experiment Data

The "solubility" of $[Al_{12}(CH_2)_{12}(CH_3)_{12}]$ (**2b**) is limited to THF, where the cluster disaggregates into several species. Three species $[(THF)Me_2Al–CH_2–AlMe_2(THF)]$ (**A**), $[MeAl(CH_2)(THF)]_3$ (**B**), and $Me_3Al(THF)$ (**C**) are suggested. The evaluation of the DOSY experiment confirmed three species. Furthermore, the presumed structures with molecular weights match well with those calculated (see Table S1b).[3]

Table S1a. Diffusion coefficients in THF-d_8 determined via PGSE NMR experiments

Signal	Diffusion coefficient	Hydrodynamic radii (measured)	Hydrodynamic radii (calculated)	Species
0 ppm	$2.458 \cdot 10^{-9}$ m²/s	--	3.15 Å	TMS (internal reference)
-0.966 ppm (CH_3)	$2.121 \cdot 10^{-9}$ m²/s	3.65 Å	3.73 Å	**C**
-0.995 ppm (CH_3)	$1.552 \cdot 10^{-9}$ m²/s	5.12 Å	4.66 Å	**A**
-1.823 ppm (CH_2)	$1.473 \cdot 10^{-9}$ m²/s			
-1.036 ppm (CH_3)	$1.317 \cdot 10^{-9}$ m²/s	5.89 Å	5.26 Å	**B**
-1.816 ppm (CH_2)	$1.311 \cdot 10^{-9}$ m²/s			

The measured hydrodynamic radii were calculated from the PGSE experiments using the T1/T2 module of Topspin, a sample viscosity of η = 0,282 mPa·s which was derived from the diffusion coefficient of TMS in the sample and its calculated hydrodynamic radius at 298 K using the Stokes-Einstein equation $\eta = \frac{k_B \cdot T}{6 \cdot \pi \cdot r_H \cdot D}$.
The calculated hydrodynamic radii were obtained from the energy optimized geometries of the molecules and the calculated solvent accessible volumes with a probe radius of 1.4 Å.

Table S1b. Molecular weight determination *via* PGSE experiment and the method described in [3]

	Species **C** [g/mol]	Species **A** [g/mol]	Species **B** [g/mol]
Highly compacted spheres	125	248	330
Ellipsoids	137	244	311
Expanded discs	161	252	304
Merged	143	249	313
Calculated	*144*	*272*	*384*

These results together with the NMR experiments performed on the THF sample lead to the following three structures present after dissolving $[(CH_3)_{12}Al_{12}(CH_2)_{12}]$ in THF-d_8.

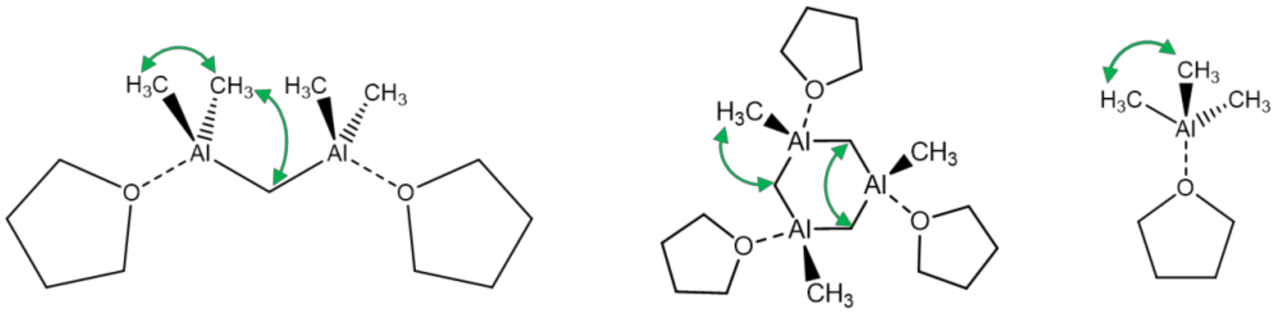

A **B** **C**

Figure S21. Structures in THF-d_8 derived by PGSE experiments (the green arrows indicate the correlations which can be seen in the HMBC spectrum (Figure S14).

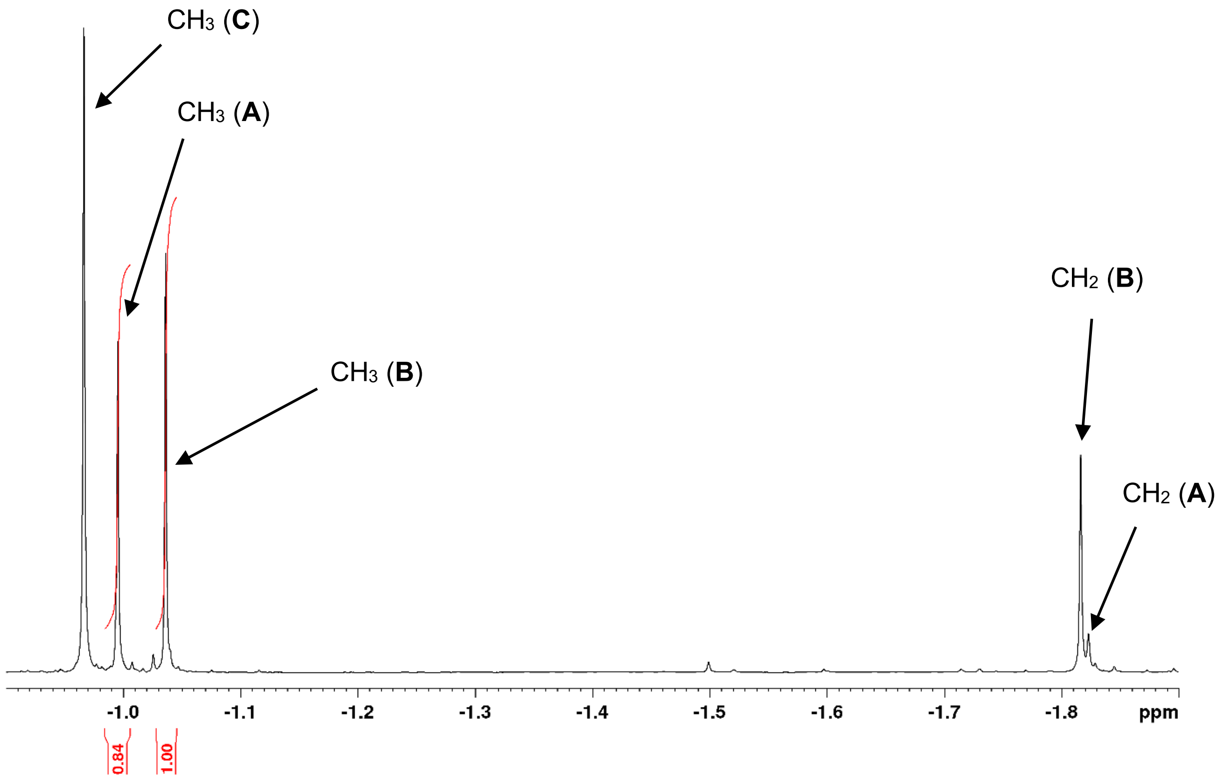

Figure S22. ^{1}H NMR spectrum of $[(CH_3)_{12}Al_{12}(CH_2)_{12}]$ (**2b**) in THF-d_8 with signal assignment and integrals.

From the methyl signals at -0.99 and -1.03 ppm for **A** and **B**, respectively, the ratio of the three species formed in THF can be calculated:

Table S2a. Ratios of the two methylene species in THF derived from the integrals of the NMR methyl signals

Signal	Species (number of CH_3 groups)	Integral	Normalized Integral	Percentage	Molar ratio (**A**:**B**)
-0.99 ppm	**A** (4)	0.84	0.63	38.7%	7:11
-1.03 ppm	**B** (3)	1	1	61.3%	

Taking into account species **C**, a percentage and molar ratio (**A**:**B**:**C**) of 19%:30%:50% and 7:11:19, respectively, is obtained.

This result correlates well with the ratio obtained by evaluating the integrals of the CH_2 groups. Due to signal overlap this can only be derived from a deconvolution of the two signals at app. -1.82 ppm:

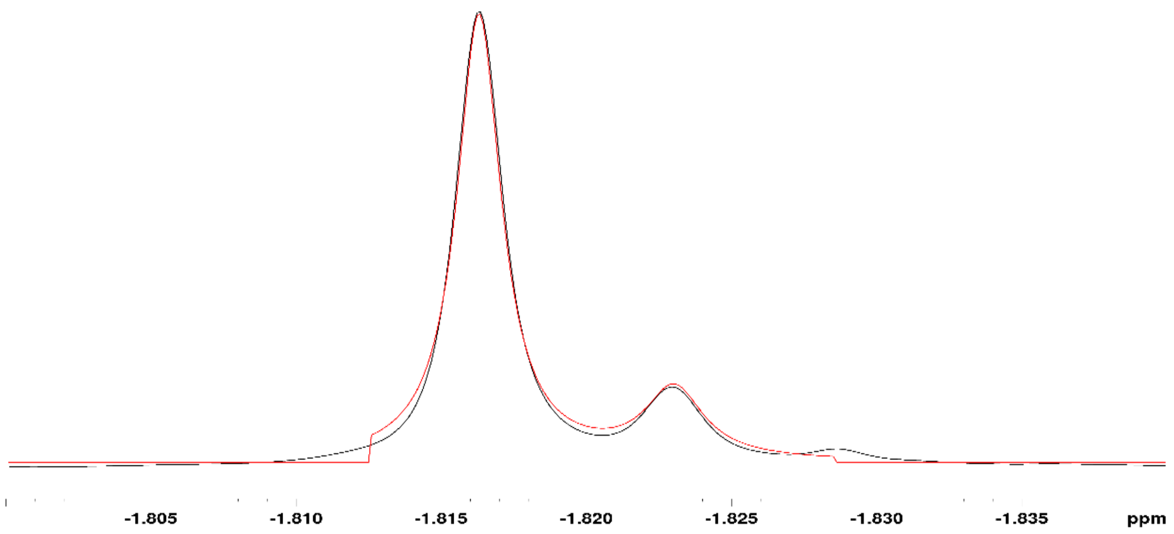

Figure S23. ^1H NMR signals of the CH_2 groups and the corresponding deconvolution result (red curve).

Table S2b. Ratios of the two species in THF derived from the integrals of the NMR methylene signals

Signal	Species (number of CH_2 groups)	Integral	Normalized integral	Percentage	Molar ratio (**A**:**B**)
-1.816 ppm	**B** (3)	4.83	1.61	38.3 %	5:8
-1.823 ppm	**A** (1)	1	1	61.7 %	

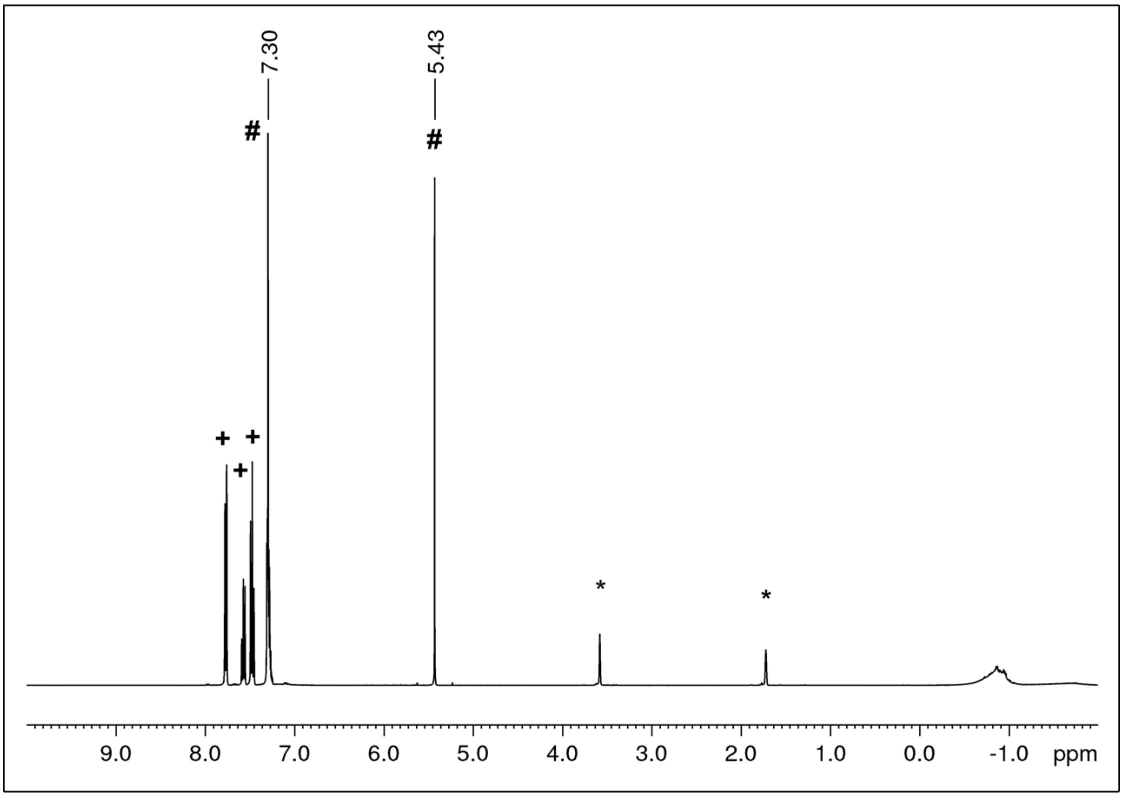

Figure S24. ^{1}H NMR spectrum (400.11 MHz) of the reaction of $[(CH_3)_{12}Al_{12}(CH_2)_{12}]$ (**2b**) with 12 equiv. benzophenone (+) in THF-d_8 (*). Product 1,1-diphenylethylene (#).

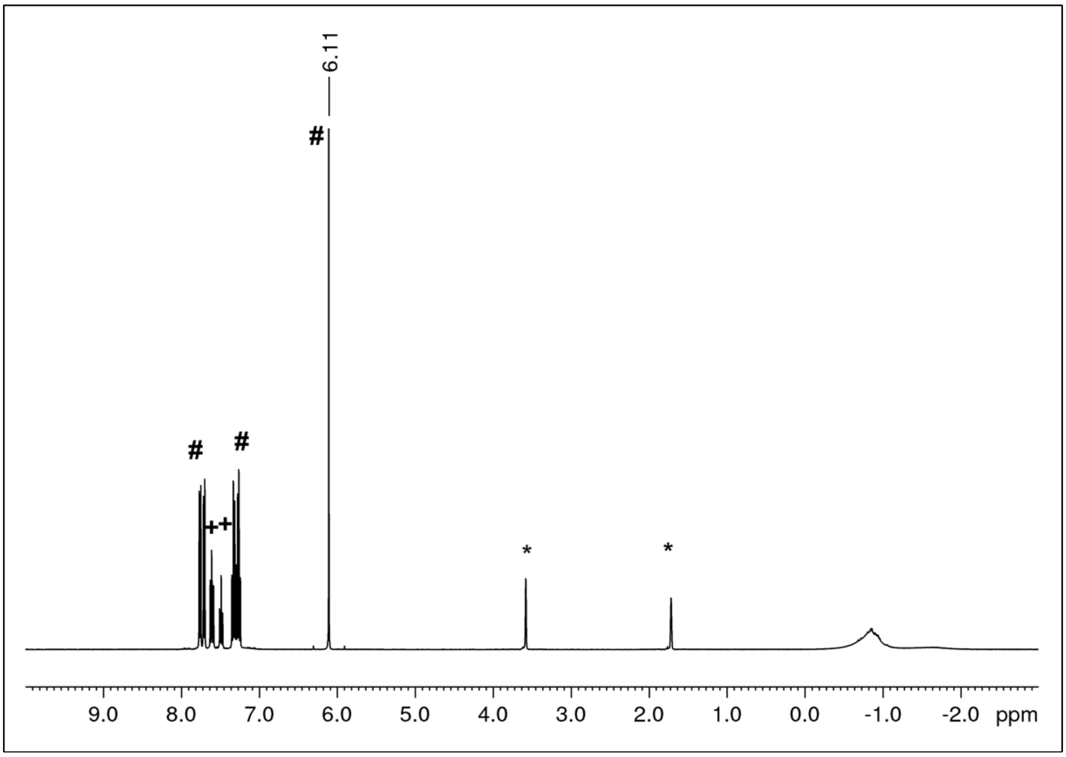

Figure S25. ^{1}H NMR spectrum (400.11 MHz) of the reaction of $[(CH_3)_{12}Al_{12}(CH_2)_{12}]$ (**2b**´) with 12 equiv. 9-fluorenone (+) in THF-d_8 (*). Product 9-methylene-fluorenone (#).

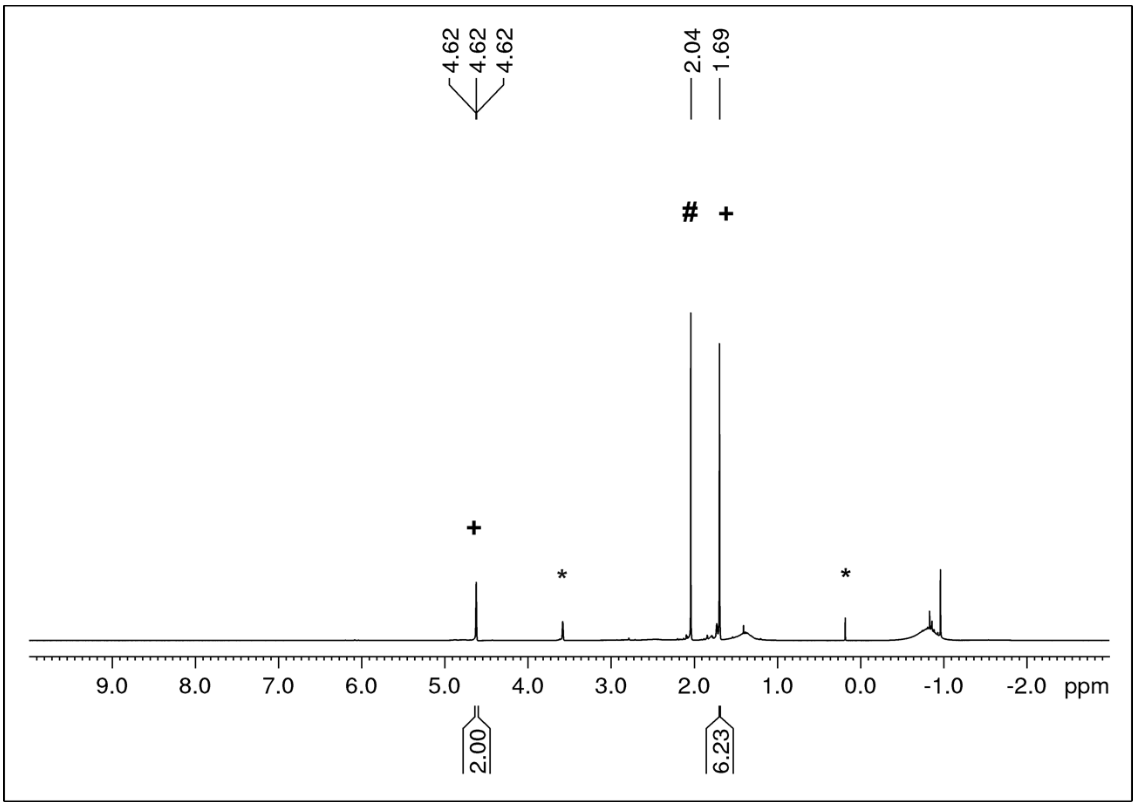

Figure S26. ^{1}H NMR spectrum (400.11 MHz) of the reaction of compound $[(CH_3)_{12}Al_{12}(\mu_3\text{-}CH_2)_{12}]$ (**2b**) with 12 equiv. acetone (#) in THF-d_8 (*). Product isobutene (+).

X-Ray Crystallography

Compound **1** was crystallized from a reaction mixture of $Cp^*_2LuAlMe_4$ and $AlMe_3$ in toluene-d_8 heated to 130 °C. Single crystals of **2a/b** were obtained by heating a suspension of $Ga_8(CH_2)_{12}$ and $AlMe_3$ in benzene to 70 °C. Single crystals were selected and coated with Paratone N (known as Parabar 10312) on a microloop. The crystals were mounted under an argon stream to prevent possible decomposition. Single crystal X-ray diffraction studies were performed on a Bruker APEX DUO instrument with an IμS microfocus sealed tube and QUAZAR optic for MoK_α radiation (λ = 0.71073 Å). The data collection strategy was determined using COSMO[4] employing ω-scans. Raw data were processed with APEX[5] and SAINT,[6] corrections for absorption were performed using SADABS.[7] The solid-state structures were solved and refined against all data by full-matrix least-squares methods on F^2 with SHELXTL[8] and Shelxle.[9] All graphics were produced by using Mercury.[10] Data of complexes **1**, **2a**, and **2b** are given in Table S3.

Table S3. Crystallographic data for compounds **1** and **2a**, and **2b**

	**1**	**2a**	**2b**
CCDC	2157627	2157626	2157628
formula	$C_{60}H_{108}Al_{10}Lu_2 \cdot C_7H_8$	$C_{24}H_{60}Al_{10.64}Ga_{1.36}$	$C_{24}H_{60}Al_{12}$
M_r [g/mol^{-1}]	1541.33	730.44	672.48
colour/shape	colourless needles	colourless needles	colourless needles
crystal dimensions [mm]	0.252 x 0.135 x 0.126	0.237 x 0.100 x 0.086	0.169 x 0.166 x 0.164
crystal system	triclinic	monoclinic	monoclinic
space group	P-1	C2/c	C2/c
a [Å]	11.7305(6)	31.188(3)	31.268(2)
b [Å]	16.3987(8)	17.1757(16)	17.1633(12)
c [Å]	20.3079(10)	21.3486(19)	21.384(3)
α [°]	73.782(2)	90	90
β [°]	85.711(2)	131.4490(10)	131.5820(10)
γ [°]	78.890(2)	90	90
V [Å^3]	3679.9(3)	8571.7(14)	8584.2(13)
Z	2	8	8
T [K]	100(2)	100(2)	100(2)
λ [Å]	0.71073	0.71073	0.71073
ρ_{calcd} [g cm^{-3}]	1.391	1.132	1.041
μ [mm^{-1}]	2.823	1.077	0.285
F (000)	1580	3072	2880
θ range [°]	1.315/28.700	1.471/28.760	2.293/30.525
unique reflections	18987	11132	13103
observed reflections (I > 2σ)	14048	8769	10187
R1/*w*R2 (I > 2σ)[a]	0.0392/0.0853	0.0315/0.0785	0.0350/0.0905
R1/*w*R2 (all data)[a]	0.0628/0.0968	0.0458/0.0853	0.0905/0.0994
GOF	1.032	1.043	1.032

[a] $R1 = \Sigma(||F_0|-|F_c||)/\Sigma|F_0|$, $F_0 > 4\sigma(F_0)$. $wR2 = \{\Sigma[w(F_0^2-F_c^2)^2/\Sigma[w(F_0^2)^2]\}^{1/2}$.

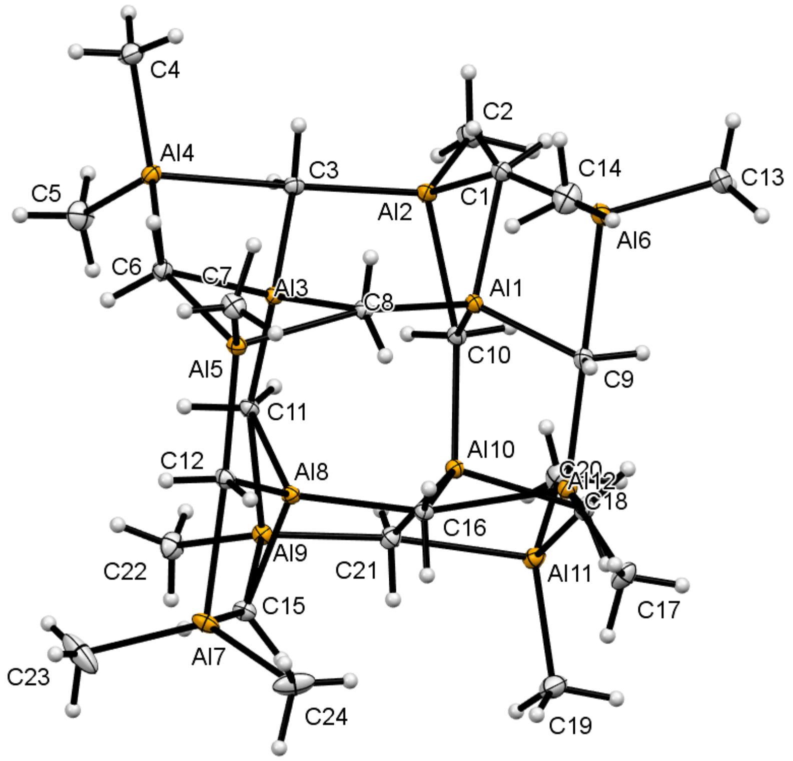

Figure S27. Crystal structure of $[(CH_3)_{12}Al_{12}(\mu_3\text{-}CH_2)_{12}]$ (**2b**). Atomic displacement parameters set at 50% probability. All hydrogen atoms except C19 were located from difference Fourier maps and refined isotropic or in some cases a constant temperature-independent multiplier of 1.5 was given.

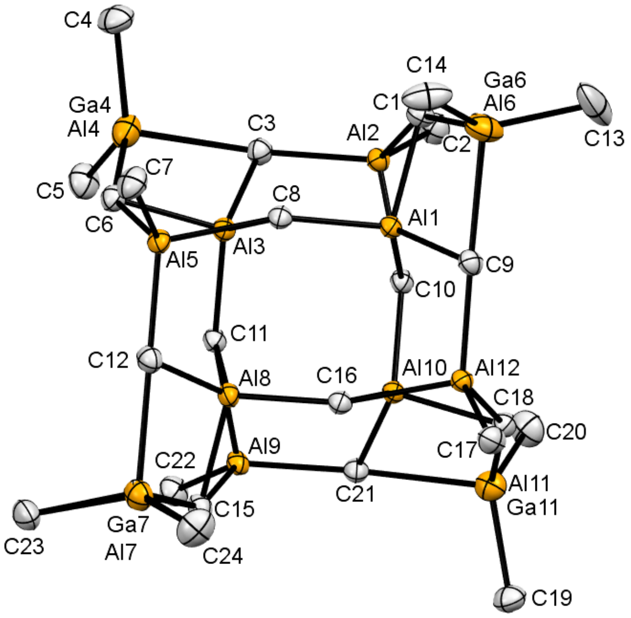

Figure S28. Crystal structure of $[(CH_3)_{12}Al_{12}(\mu_3\text{-}CH_2)_{12}]$ (**2a**). Atomic displacement parameters set at 50% probability. Hydrogen atoms are omitted for clarity.

IR Spectra

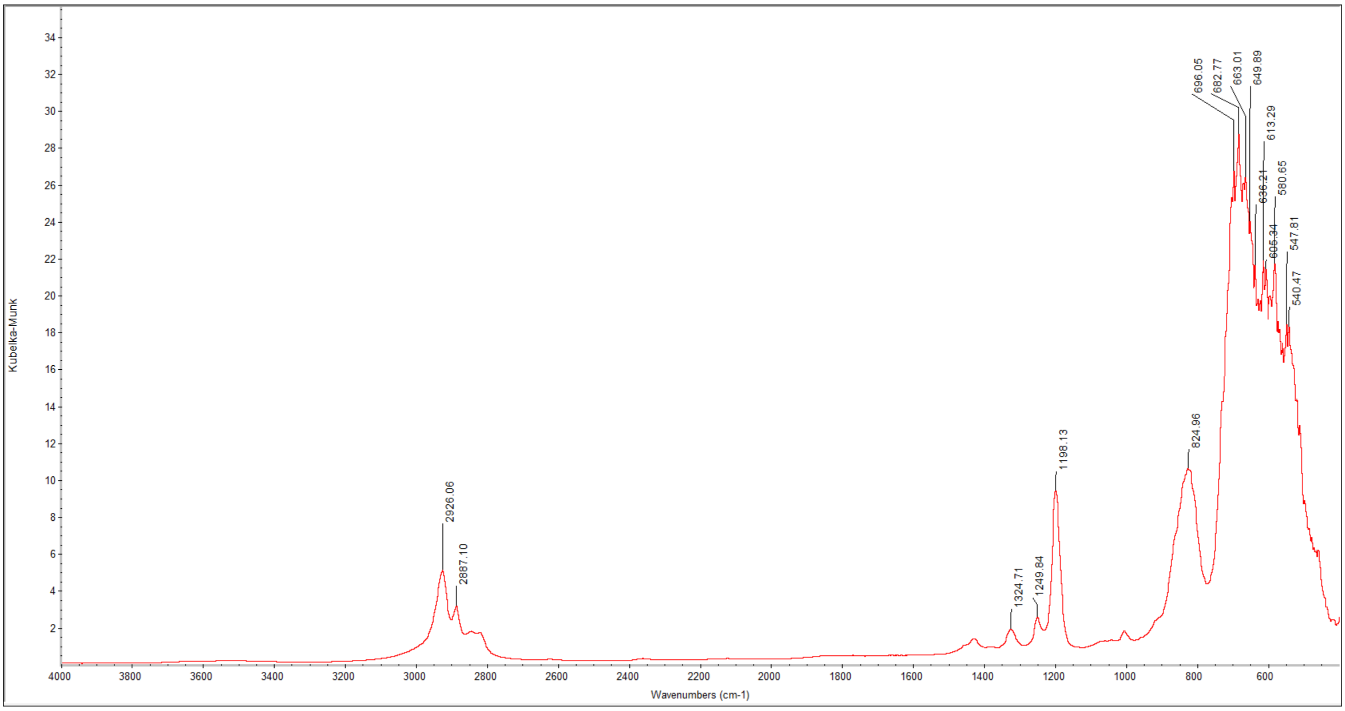

Figure S29. DRIFT spectrum of compound **2b**.

References

[1] V. M. Birkelbach, F. Kracht, H. M. Dietrich, C. Stuhl, C. Maichle-Mössmer, R. Anwander, *Organometallics* **2020**, *39*, 3490-3504.
[2] M. Bonath, C. Maichle-Mössmer, P. Sirsch, R. Anwander, *Angew. Chem. Int. Ed.* **2019**, *58*, 8206-8210; *Angew. Chem.* **2019**, *131*, 8290-8294.
[3] R. Neufeld, D. Stalke, *Chem. Sci.* **2015**, *6*, 3354-3364.
[4] *COSMO v. 1.61*, Bruker AXS Inc., Madison, WI, 2012.
[5] APEX3 v2019.11-0, Bruker AXS Inc., Madison, WI, 2019.
[6] *SAINT V8.40B, Bruker Nano, Inc., 2019.*
[7] *SADABS*: L. Krause, R. Herbst-Irmer, G. M. Sheldrick, D. Stalke, *J. Appl. Cryst.* **2015**, *48***,** 3-10.
[8] *SHELXT*: G. M. Sheldrick, *Acta Cryst.* **2015**, *A71*, 3-8.
[9] SHELXLE: C. B. Huebschle, G. M. Sheldrick, B. Dittrich, *J. Appl. Crystallogr.* **2011**, *44*, 1281-1284.
[10] Mercury CSD 2.0: C. F. Macrae, I. J. Bruno, J. A. Chisholm, P. R. Edgington, P. McCabe, E. Pidcock, L. Rodriguez-Monge, R. Taylor, J. van de Streek, P. A. Wood, *J. Appl. Cryst.* **2008**, *41*, 466-470.
